# Kaempferol attenuates particle-induced osteogenic impairment by regulating ER stress *via* the IRE1α–XBP1s pathway

**DOI:** 10.1016/j.jbc.2024.107394

**Published:** 2024-05-18

**Authors:** Xin Yu, Zhengrong Ren, Yuxiang Wang, Guodong Yuan, Jianlun Hu, Lin Song, Cheng Pan, Kangkang Feng, Yuqiao Liu, Longgang Shao, Li Zhang, Jinjuan Wang, Jianning Zhao, Nirong Bao, Zhongyang Sun

**Affiliations:** 1 Department of Orthopedics, Nanjing Jinling Hospital, Affiliated Hospital of Medical School, Nanjing University, Nanjing, China; 2 State Key Laboratory of Pharmaceutical Biotechnology, School of Life Sciences, Nanjing University, Nanjing, China; 3 Department of Orthopedics, The Second Hospital of Nanjing, Nanjing University of Chinese Medicine, Nanjing, China; 4 State Key Laboratory of Pharmaceutical Biotechnology, Jiangsu Key Laboratory of Molecular Medicine, Medical School, Nanjing University, Nanjing, China; 5 Department of Thoracic Surgery, The First Affiliated Hospital of Nanjing Medical University, Nanjing, China; 6 Medical Information Data Bank, Nanjing Jinling Hospital, Affiliated Hospital of Medical School, Nanjing University, Nanjing, China; 7 Department of Emergency Medicine, The Second Affiliated Hospital of Nanjing University of Chinese Medicine, Nanjing, China; 8 Department of Prosthodontics, Nanjing Stomatological Hospital, Affiliated Hospital of Medical School, Nanjing University, Nanjing, China; 9 Department of Pharmacy, Nanjing First Hospital, Nanjing Medical University, Nanjing, China; 10 Department of Orthopedics, Air Force Hospital of Eastern Theater, Anhui Medical University, Nanjing, China

**Keywords:** Kaempferol, IRE1α/XBP1s, aseptic loosening, osteolysis, ER stress, osteoblast

## Abstract

Periprosthetic osteolysis and subsequent aseptic loosening are the primary causes of failure following total joint arthroplasty. Wear particle–induced osteogenic impairment is recognized as an important contributing factor in the development of osteolysis, with endoplasmic reticulum (ER) stress emerging as a pivotal underlying mechanism. Hence, searching for potential therapeutic targets and agents capable of modulating ER stress in osteoblasts is crucial for preventing aseptic loosening. Kaempferol (KAE), a natural flavonol compound, has shown promising osteoprotective effects and anti-ER stress properties in diverse diseases. However, the influence of KAE on ER stress-mediated osteogenic impairment induced by wear particles remains unclear. In this study, we observed that KAE effectively relieved TiAl_6_V_4_ particles–induced osteolysis by improving osteogenesis in a mouse calvarial model. Furthermore, we demonstrated that KAE could attenuate ER stress-mediated apoptosis in osteoblasts exposed to TiAl_6_V_4_ particles, both *in vitro* and *in vivo*. Mechanistically, our results revealed that KAE mitigated ER stress-mediated apoptosis by upregulating the IRE1α–XBP1s pathway while concurrently partially inhibiting the IRE1α-regulated RIDD and JNK activation. Collectively, our findings suggest that KAE is a prospective therapeutic agent for treating wear particle–induced osteolysis and highlight the IRE1α–XBP1s pathway as a potential therapeutic target for preventing aseptic loosening.

Aseptic loosening is a common complication of artificial joint replacement, which is the main reason for implant failure and revision surgery (1, 2). The generation of wear particles during long-term use of prosthesis is the primary triggering cause of aseptic loosening (3). Osteoblasts play a crucial role in the entire process of bone remodeling, and any changes in their quantity and functionality directly impact the occurrence and progression of aseptic loosening (4, 5). Wear particles have been shown to have detrimental effects on osteoblast survival, proliferation, and function, leading to decreased peri-prosthetic bone formation and improper bone remodeling (4, 5, 6). Previous studies by our laboratory and others have demonstrated that endoplasmic reticulum (ER) stress mediates wear particle–induced osteoblast apoptosis and osteolysis (7, 8, 9). Therefore, targeting ER stress-mediated osteoblast apoptosis holds potential as a therapeutic strategy for alleviating periprosthetic osteolysis and aseptic loosening.

The ER, essential in eukaryotic cells, governs protein synthesis, folding, and processing (10). Disruption of ER homeostasis leads to the accumulation of unfolded or misfolded proteins, triggering ER stress and activating the unfolded protein response (UPR) (11, 12). While UPR activation initially promotes adaptive ER remodeling and cell survival, prolonged activation under chronic ER stress activates pro-apoptotic programs, leading to cell death (13, 14). The UPR involves three signaling cascades initiated by ER stress-sensing proteins: IRE1α, PERK, and ATF6, with IRE1α being the most evolutionarily conserved and possessing kinase and RNase activities (15). Under ER stress, IRE1α activates its RNase activity, splicing XBP1 mRNA to produce the active form, XBP1s, which induces adaptive genes to alleviate ER stress and restore proteostasis (16, 17). However, prolonged IRE1α RNase activity may lead to promiscuous degradation of ER-localized mRNA *via* regulated IRE1α-dependent decay (RIDD), a mechanism associated with ER stress-associated apoptosis (11, 18, 19). Moreover, extended UPR activation allows IRE1α to recruit TRAF2 and initiate c-Jun N-terminal kinase (JNK)-mediated pro-apoptotic signaling (11, 16, 20). Selective activation of the IRE1α–XBP1s pathway is a promising strategy to mitigate pathologic imbalances in ER proteostasis implicated in diverse diseases (16, 17, 18). Additionally, the IRE1α–XBP1s pathway crucially regulates osteoblast differentiation, actively promoting the expression of osteoblast-related genes (21, 22, 23). Hence, targeting the IRE1α–XBP1s pathway holds potential as a therapeutic approach for preventing and treating aseptic loosening.

Kaempferol (KAE), a natural flavonoid found in various dietary sources and traditional herbal medicine, exhibits a diverse range of pharmacological properties, including anti-inflammatory, antioxidant, anticancer, and anti-aging activities (24, 25, 26). Recently, accumulating evidence has indicated that KAE exhibits promising bone-forming and osteoprotective effects on the skeleton (27, 28, 29, 30, 31, 32, 33). In our previous study, we found that KAE could ameliorate wear particle–induced osteolysis by reducing inflammatory responses and limiting excessive osteoclast formation (34). However, the specific impact of KAE on particle-induced osteoblast apoptosis and osteogenic reduction remains unknown. It is known that ER stress mediates such osteoblast apoptosis and osteogenic reduction (7, 8, 9). KAE has demonstrated potential in promoting cell survival and maintaining cellular function, particularly by modulating ER stress (35, 36, 37, 38, 39, 40, 41). Flavonol compounds, exemplified by quercetin, have been reported to potentially interact with the Q-site, a ligand-binding pocket located at the back-to-back dimer interface of IRE1α′s kinase extension nuclease domain, thereby activating its RNase activity and enhancing the cleavage of XBP1 mRNA (42). It is noteworthy that KAE is a natural flavonol with structural similarities to quercetin, suggesting a potential involvement of the IRE1α–XBP1s pathway in mediating KAE’s ability to alleviate ER stress and improve ER proteostasis (11, 42). Additionally, Abdullah *et al.* have previously reported that KAE binds to the ATP-binding site of IRE1α, thus activating its RNase activity and inducing differentiation of neuroblastoma cells *via* IRE1α–XBP1s pathway (43). Considering these findings, the potential of KAE in alleviating ER stress-associated osteoblast apoptosis and osteogenic reduction, particularly in the context of wear particle–induced osteolysis, holds considerable promise and warrants further investigation.

In the present study, we conducted a systematic evaluation of the pharmacological effects of KAE in the mouse calvarial osteolysis model and osteoblast exposed to particles, aiming to explore its impact on ER stress-mediated apoptosis and osteogenic reduction, while trying to elucidate its underlying mechanisms of action. Through this study, we anticipate gaining a deeper insight into the regulatory role of KAE in ER stress and osteogenic function, providing a theoretical basis for the development of novel strategies and interventions for the prevention and treatment of periprosthetic osteolysis and aseptic loosening.

## Results

### KAE treatment mitigated TiPs-induced bone destruction and improved bone formation in a mouse calvarial osteolysis model

**Figure 1:**
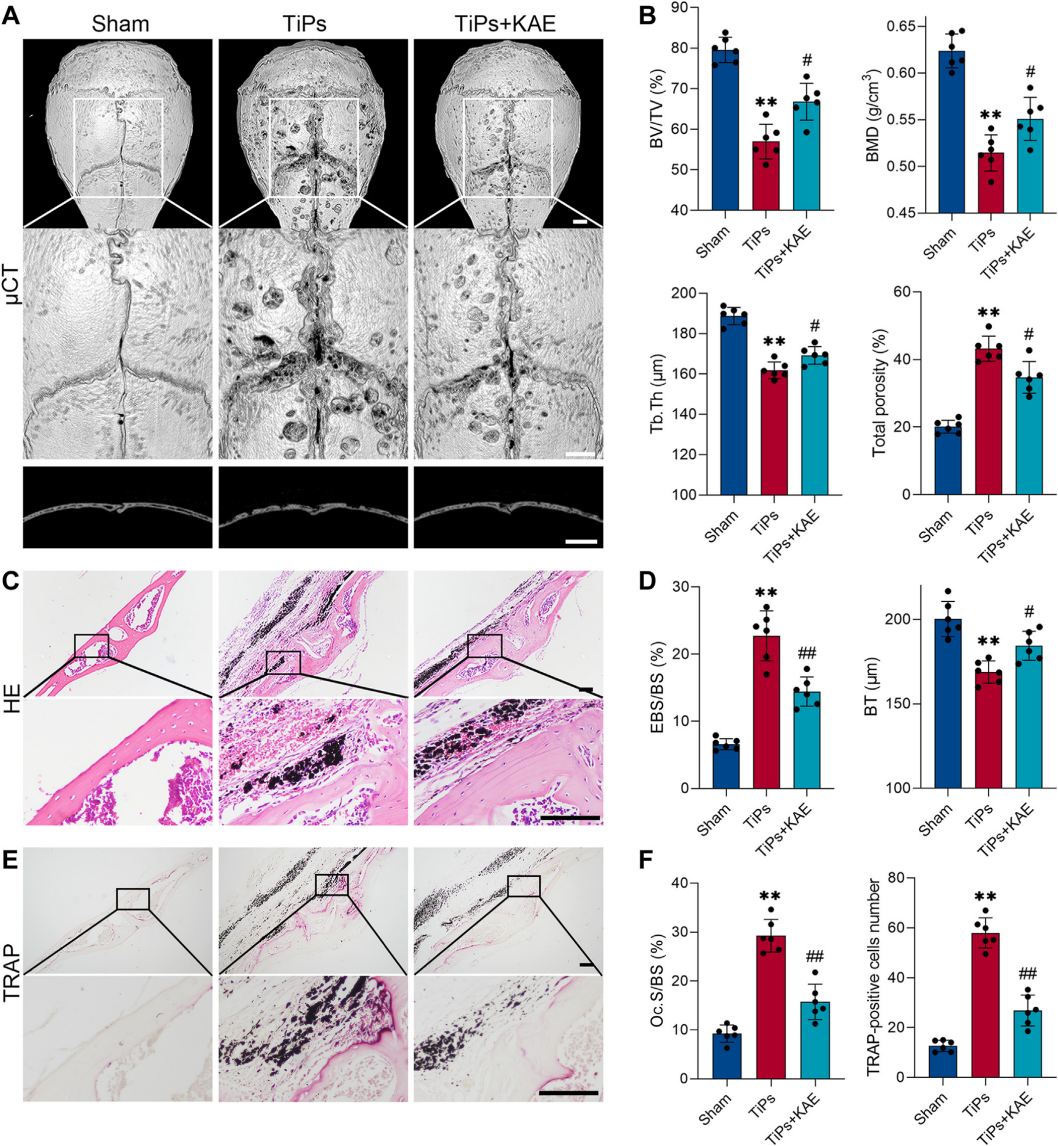
**KAE treatment relieved TiPs-induced osteolysis in a mouse calvarial model.***A*, representative micro-CT (μCT) three-dimensional reconstructed images (*top* and *middle*) and cross-sectional images (*bottom*) of mouse calvaria in each group. Scale bar represents 1 mm. *B*, quantitative analysis of bone morphometric parameters, including BV/TV (%), BMD (g/cm^3^), Tb.Th (μm), and total porosity (%), *n* = 6. *C*, representative H&E staining images of mouse calvarial sections from each group. Scale bar represents 100 μm. *D*, quantitative measurement of eroded bone surface per bone surface (EBS/BS, %) and bone thickness (BT, μm) in each group. *n* = 6. *E*, representative TRAP staining images of mouse calvarial sections from each group. Scale bar represents 100 μm. *F*, quantitative measurement of osteoclast surface per bone surface (Oc.S/BS, %) and TRAP-positive cell number in each group. *n* = 6. All values are presented as mean ± SD. One-way ANOVA. ∗∗*p* < 0.01 *versus* the sham group. ^#^*p* < 0.05 and ^##^*p* < 0.01 *versus* the TiPs group. BMD, bone mineral density; BV/TV, bone volume to total volume; KAE, Kaempferol; micro-CT, micro-computed tomography; Tb.Th, total porosity and trabecular thickness; TiPs, TiAl_6_V_4_ particles; TRAP, tartrate-resistant acid phosphatase.

To determine the effects of KAE on TiAl_6_V_4_ particles (TiPs)-induced osteolysis, miro-CT scanning, H&E, and tartrate-resistant acid phosphatase (TRAP) staining were carried out. The micro-computed tomography (micro-CT) findings demonstrated a significant alleviation of TiPs-induced bone destruction upon treatment with KAE (Fig. 1*A*). Quantitative analysis of bone morphometric parameters, including bone volume-to-total volume, bone mineral density, total porosity and trabecular thickness, and total porosity, consistently confirmed the mitigating effects of KAE on TiPs-induced bone loss (Fig. 1*B*). H&E staining exhibited a marked reduction in TiPs-induced bone erosion with KAE treatment, evidenced by a decrease in the ratio of eroded bone surface to bone surface and alleviation of bone thickness reduction (Fig. 1, *C* and *D*). Moreover, TRAP staining revealed that KAE notably suppressed the increase in osteoclast number and osteoclast surface area per bone surface induced by TiPs stimulation (Fig. 1, *E* and *F*), consistent with our prior research findings (34).

**Figure 2:**
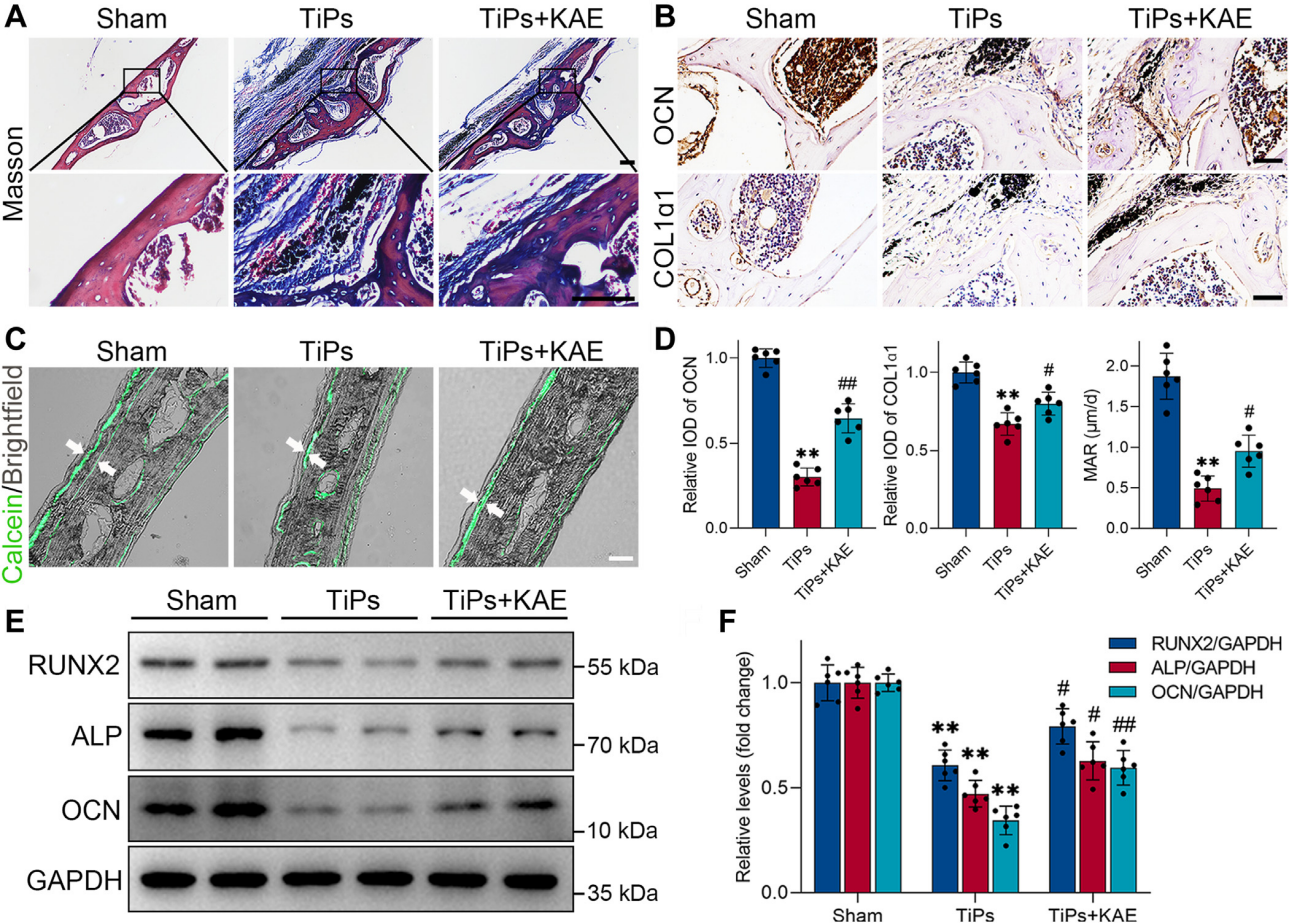
**KAE treatment alleviated TiPs-induced reduction in bone formation *in vivo*.***A*, representative Masson’s trichrome staining of mouse calvarial sections from each group. Scale bar represents 100 μm. *B*, representative images of immunohistochemical staining for OCN (*upper* images) and COL1α1 (*lower* images). Scale bar represents 50 μm. *C*, representative images of calcein double labeling with a 10-days interval. Scale bar represents 50 μm. *D*, quantitative immunohistochemical analysis for OCN and COL1α1, and measurement of the average periosteum mineral apposition rates (MAR, μm/d) in each group, *n* = 6. *E*-*F*, Western blot analysis of osteogenic markers (RUNX2, ALP, OCN) in mouse calvarial bone tissue samples per group. *n* = 6. All values are presented as mean ± SD. One-way ANOVA. ∗∗*p* < 0.01 *versus* the sham group. ^#^*p* < 0.05 and ^##^*p* < 0.01 *versus* the TiPs group. ALP, alkaline phosphatase; KAE, Kaempferol; OCN, osteocalcin; RUNX2, Runt-related transcription factor 2; TiPs, TiAl_6_V_4_ particles.

To assess bone formation in mouse calvaria, a comprehensive set of histological and molecular assays were performed, including Masson’s trichrome staining, immunohistochemical staining, calcein double labeling, and Western blotting. The Masson’s trichrome staining results distinctly exhibited a higher presence of newly formed bone collagen fibers in the KAE-treated group when compared to the model group (Fig. 2*A*). Immunohistochemical results further highlighted the favorable effects of KAE treatment on bone formation by revealing an upregulation in the expression of key osteogenic markers, namely osteocalcin (OCN) and COL1α1 (Fig. 2, *B* and *D*). Moreover, the calcein fluorescent double-labeling assay provided compelling evidence of KAE’s potential to promote bone formation, as evidenced by an increased mineral apposition rate (Fig. 2, *C* and *D*). Consistent with these findings, Western blot analysis confirmed the enhanced expression levels of osteogenic markers (Runt-related transcription factor 2 (RUNX2), alkaline phosphatase (ALP), and OCN) in the KAE-treated group when compared to the model group, further supporting the beneficial effects of KAE on bone formation (Fig. 2, *E* and *F*).

To further evaluate the *in vivo* biosafety of KAE, we examined its impact on body weight, serum biochemical parameters, and major organ histopathology. As shown in Fig. S1, *C* and *D*, KAE did not cause any adverse effects on mouse body weight and liver and renal function. Moreover, there were no significant differences observed in the H&E staining images of major organs (heart, liver, spleen, lung, and kidney) across all groups (Fig. S1*E*). These findings collectively indicated that KAE exhibited favorable biosafety *in vivo*.

### KAE treatment relieved ER stress-mediated apoptosis in osteoblasts exposed to TiPs *in vitro* and *in vivo*

The increase of osteoblast apoptosis triggered by wear particles results in decreased bone formation around the prosthesis, which is an etiology for periprosthetic osteolysis and aseptic loosening (3, 4, 5). To investigate the impact of the prepared TiPs on osteoblast cell viability and apoptosis levels *in vitro*, we conducted both the cell counting kit-8 (CCK-8) assay and flow cytometry assay. As shown in Fig. S2, *A*–*C*, TiPs treatment decreased the cell viability in a dose-dependent manner, concurrently accompanied by a corresponding increase in the apoptosis rate. Subsequently, we proceeded to examine the effects of KAE on TiPs-induced cytotoxicity in osteoblasts. Although KAE led to a slight decrease in osteoblast cell viability at a concentration of 20 μM, no significant effects on cell viability were observed at the dose (10 μM), which was chosen for subsequent *in vitro* experiments (Fig. S2*D*). As shown in Fig. S2*E*, KAE significantly mitigated the cytotoxic effects of TiPs on osteoblasts. Notably, the most pronounced enhancement in cell viability was observed at the concentration of 10 μM KAE, without affecting the apoptosis levels of osteoblasts (Fig. S2, *E*–*G*).

**Figure 3:**
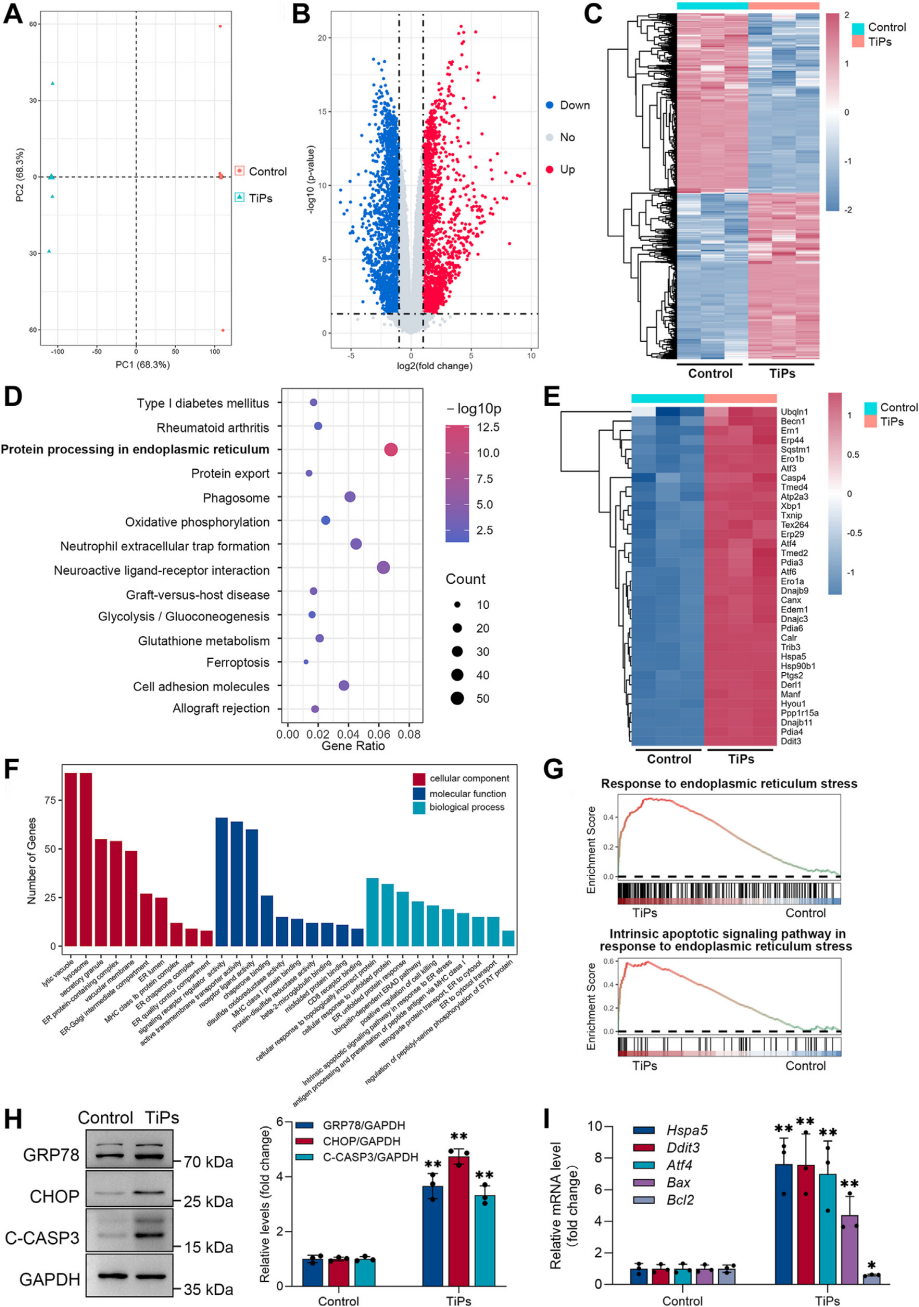
**ER stress mediates TiPs-induced osteoblast apoptosis.***A*, principal component analysis (PCA) plot of the RNA-seq datasets obtained from osteoblasts treated with or without TiPs (50 μg/ml) for 24 h. *B*, volcano plot illustrating the differential gene expression in osteoblasts treated with TiPs compared to the control group, with significantly upregulated genes highlighted in *red* and significantly downregulated genes in *blue*. *C*, heat map showing differentially expressed genes in osteoblasts treated with or without TiPs (50 μg/ml) for 24 h. *D*, KEGG enrichment analysis of differentially expressed genes. *E*, heat map illustrating the differential expression of genes related to ER stress in the control and TiPs-treated group. *F*, gene Ontology (GO) analysis of cellular components, molecular functions, and biological processes of differentially expressed genes. *G*, gene set enrichment analysis (GSEA) revealed that the GO items related to the response to ER stress and intrinsic apoptotic signaling pathway in response to ER stress were enriched in the TiPs group compared to the Control group. *H*, Western blot analysis of ER stress markers, including the ER chaperone GRP78 and the ER stress-related pro-apoptotic transcription factor CHOP and the apoptosis marker C-CASP3 in osteoblasts treated with or without TiPs (50 μg/ml) for 24 h. *I*, qRT-PCR analysis of mRNA levels of genes associated with ER stress–mediated apoptosis in osteoblasts with or without TiPs (50 μg/ml) for 24 h. All values are presented as mean ± SD. Unpaired Students’s *t* test, ∗*p* < 0.05 and ∗∗*p* < 0.01 *versus* the control group. KEGG, kyoto encyclopedia of genes and genomes; TiPs, TiAl_6_V_4_ particles; qRT-PCR, quantitative reverse transcription-PCR.

Previous studies have established that ER stress plays an important role in mediating wear particle–induced osteoblast apoptosis and osteogenic reduction (7, 8, 9). To further substantiate the involvement of ER stress in TiPs-induced osteogenic impairment, analysis of differentially expressed genes (DEGs) in osteoblasts treated with or without TiPs was performed using RNA-seq. Principal component analysis results revealed that the two groups were well-separated in terms of gene expression profiles (Fig. 3*A*). As shown in Figure 3, *B* and *C*, a total of 4564 differentially expressed genes were identified, with 48.53% upregulated and 51.47% downregulated. Kyoto Encyclopedia of Genes and Genomes and Gene Ontology (GO) enrichment analyses revealed that these DEGs were primarily involved in pathways and biological processes related to ER protein processing and UPR, and the heatmap displayed a significant upregulation of genes associated with ER stress induced by TiPs (Fig. 3, *D*–*F*). Furthermore, gene set enrichment analysis illustrated the significant enrichment of GO items related to the response to ER stress and intrinsic apoptotic signaling pathway in response to ER stress in the TiPs group, further supporting the strong association between ER stress and wear particle–induced osteoblast apoptosis (Fig. 3*G*). To validate these observations, we examined the expression levels of ER stress and apoptosis markers in osteoblasts. As shown in Figure 3*H*, Western blot analysis revealed a significant elevation in the protein expression levels of ER chaperone GRP78, ER stress-related pro-apoptotic transcription factor CHOP, and apoptosis marker C-CASP3 following TiPs exposure. Moreover, quantitative reverse transcription-PCR (qRT-PCR) analysis showed that TiPs exposure upregulated the expression of ER stress-related genes (*Hspa5*, *Ddit3*, *Atf4*) and apoptosis-related gene (*Bax*) while downregulating the expression of anti-apoptotic gene *Bcl2* (Fig. 3*I*). These findings provide further support for the involvement of ER stress in TiPs-induced osteogenic impairment and highlight the potential mechanism underlying TiPs-induced osteoblast apoptosis.

**Figure 4:**
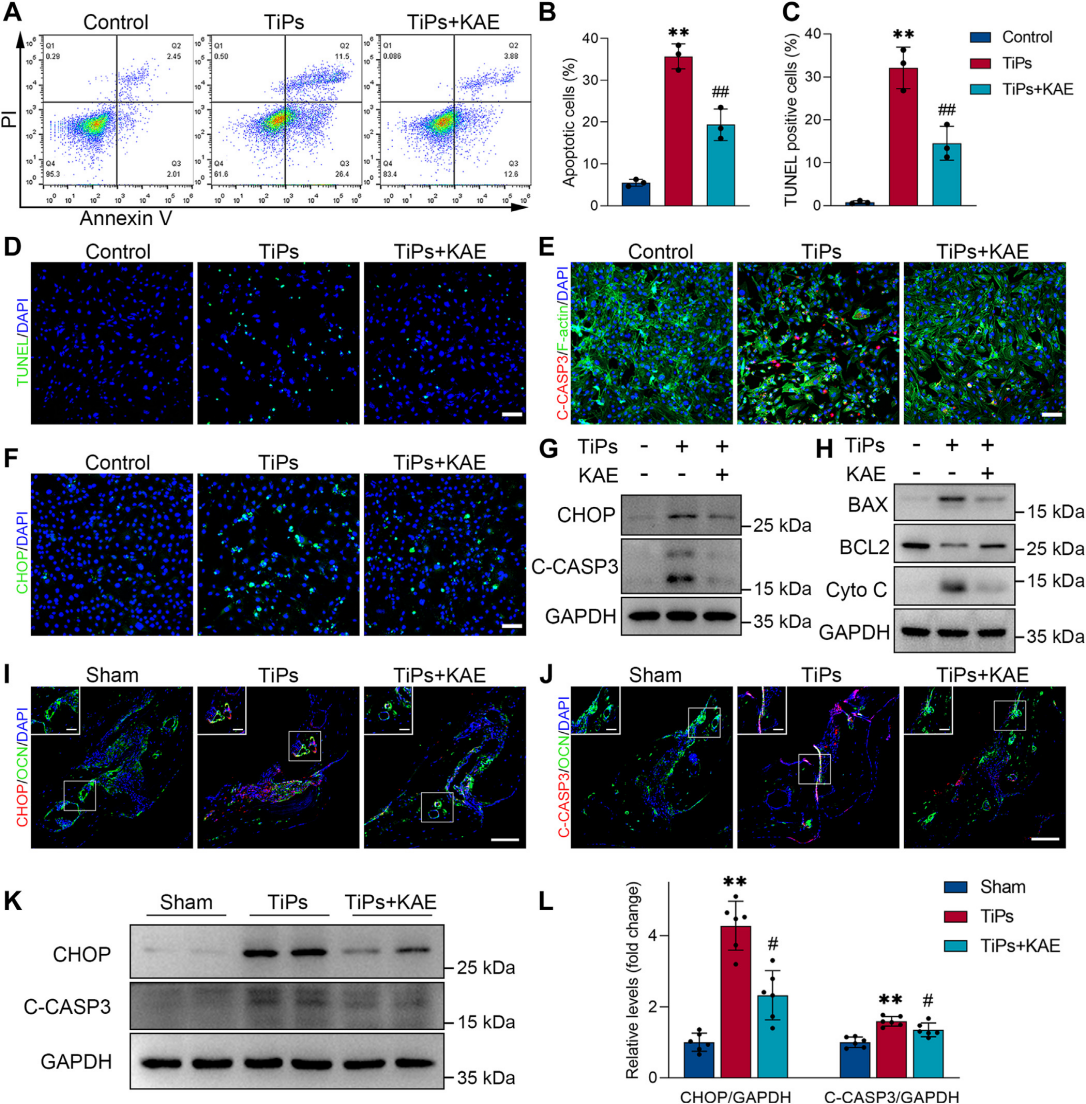
**KAE treatment inhibited ER stress-mediated apoptosis in osteoblasts exposed to TiPs *in vitro* and *in vivo*.***A* and *B*, flow cytometry analysis of apoptosis using Annexin V/PI staining. *n* = 3. *C* and *D*, representative images of TUNEL staining and the percentage of TUNEL-positive cells in each group. *n* = 3. Scale bar represents 100 μm. *E*, representative images of immunofluorescence staining; *red* (C-CASP3), *green* (F-actin), and *blue* (nuclei). Scale bar represents 100 μm. *F*, representative images of immunofluorescence staining for the ER stress-related pro-apoptotic transcription factor CHOP. Scale bar represents 100 μm. *G*, Western blots of CHOP and the apoptosis marker C-CASP3 in osteoblasts following treatment for 24 h with medium, TiPs (50 μg/ml), or TiPs + KAE (10 μM). *H*, Western blots of BAX, BCL2, and Cyto C in osteoblasts following treatment for 24 h with medium, TiPs, or TiPs + KAE. *I*, representative images of immunofluorescence double staining for CHOP (*red*) and OCN (*green*) in histological sections of the mouse calvaria per group. Scale bar represents 100 μm; scale bar (enlarged) represents 20 μm. *J*, representative images of immunofluorescence double staining for C-CASP3 (*red*) and OCN (*green*) in histological sections of the mouse calvaria per group. Scale bar represents 100 μm; scale bar (enlarged) represents 20 μm. *K* and *L*, Western blot analysis of the ER stress-mediated apoptosis markers (CHOP, C-CASP3) in mouse calvarial bone tissue samples per group. *n* = 6. All values are presented as mean ± SD. One-way ANOVA. ∗∗*p* < 0.01 *versus* the control group or the sham group. ^#^*p* < 0.05 and ^##^*p* < 0.01 *versus* the TiPs group. Cyto C, cytochrome C; KAE, Kaempferol; OCN, osteocalcin; TiPs, TiAl_6_V_4_ particles; TUNEL, TdT-mediated dUTP nick end-labeling.

To investigate the effects of KAE on TiPs-induced osteoblast apoptosis *in vitro*, flow cytometry and TdT-mediated dUTP nick end-labeling (TUNEL) staining assays were performed. As shown in Figure 4, *A*–*D*, KAE treatment markedly mitigated the elevation of the apoptotic rate induced by TiPs. Subsequently, we utilized immunofluorescence staining and Western blot analysis to evaluate the expression levels of C-CASP3 and CHOP in osteoblasts (7, 11, 44). The results revealed that TiPs exposure led to F-actin cytoskeleton shrinkage, nuclear fragmentation, and an increase in C-CASP3 and CHOP expression levels. However, treatment with KAE notably attenuated these TiPs-induced alterations, effectively mitigating TiPs-induced ER stress and apoptosis in osteoblasts (Figs. 4, *E*–*G* and S3, *A* and *B*). Moreover, KAE treatment also downregulated the expression levels of pro-apoptotic markers (BAX and Cytochrome C), while concurrently enhancing the expression of the anti-apoptotic marker BCL2 (Figs. 4*H*, S3*C*). Additionally, *in vivo* immunostaining and Western blot analysis provided further confirmation that KAE inhibited ER stress-mediated apoptosis in osteoblasts exposed to TiPs (Fig. 4*I*–*L*).

### KAE treatment rescued TiPs-induced osteogenic inhibition in osteoblasts *in vitro*

**Figure 5:**
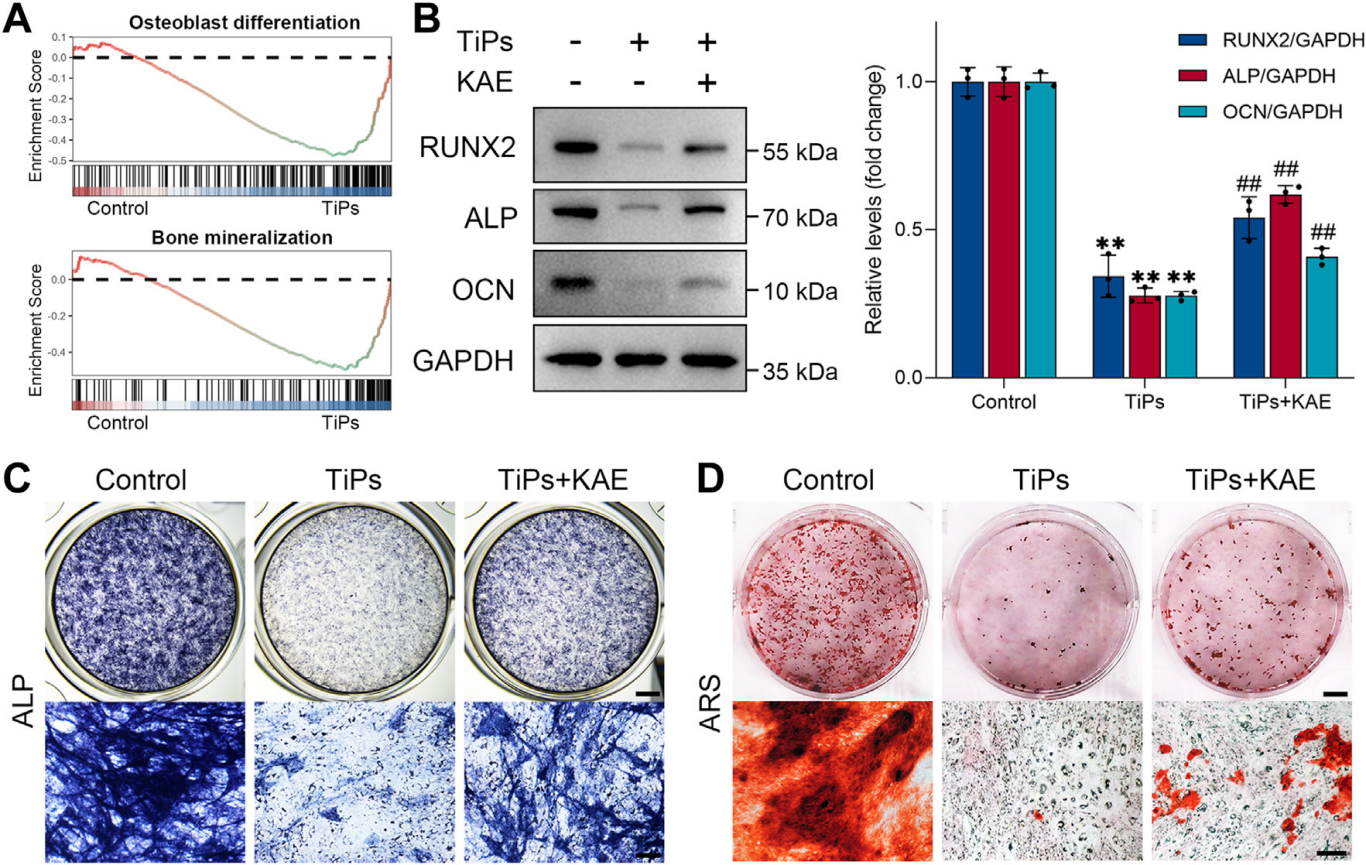
**KAE treatment attenuated TiPs-induced impairment of osteogenesis *in vitro*.***A*, GSEA analysis revealed that the GO items related to osteoblast differentiation and bone mineralization were enriched in the TiPs group compared to the control group. *B*, Western blot analysis of osteogenic markers (RUNX2, ALP, and OCN) in osteoblasts treated with different interventions after 3 days of osteogenic induction. *n* = 3. *C*, representative images of ALP staining for osteoblasts in each group. Scale bar represents 2 mm (*top*), 100 μm (*bottom*). *D*, representative images of Alizarin red S (ARS) staining for osteoblasts in each group. Scale bar represents 5 mm (*top*), 250 μm (*bottom*). All values are presented as mean ± SD. One-way ANOVA. ∗∗*p* < 0.01 *versus* the control group, ^##^*p* < 0.01 *versus* the TiPs group. ALP, alkaline phosphatase; GO, gene ontology; GSEA, gene set enrichment analysis; KAE, Kaempferol; OCN, osteocalcin; RUNX2, Runt-related transcription factor 2; TiPs, TiAl_6_V_4_ particles.

Gene set enrichment analysis of RNA-seq data demonstrated significant enrichment of GO items associated with the negative regulation of osteoblast differentiation and bone mineralization in the TiPs group, indicating the inhibitory effects of TiPs on osteoblast activity (Fig. 5*A*). Previous studies have reported the osteogenic potential of KAE, suggesting its ability to enhance osteoblast activity *in vitro* (32, 45). This underscores the potential of KAE as a therapeutic agent to counteract the detrimental effects induced by TiPs on osteoblast activity. To validate the osteogenic potential of KAE, we conducted experiments to investigate its effects on osteoblast proliferation, differentiation, and mineralization. As shown in Fig. S4, *A* and *B*, KAE demonstrated a dose-dependent promotion of osteoblast proliferation, differentiation, and mineralization. Moreover, Western blot analysis showed that KAE upregulated the expression levels of osteogenesis-related proteins, including RUNX2, ALP, and OCN, further supporting its role in promoting osteoblast activity (Fig. S4, *C* and *D*).

To further evaluate the influence of KAE on the osteogenic function of osteoblasts exposed to TiPs, a comprehensive set of assays was conducted, including Western blot analysis of osteogenic markers, ALP staining, and Alizarin red S (ARS) staining. As shown in Figure 5*B*, KAE distinctly restored the osteogenic potential of osteoblasts exposed to TiPs, as evidenced by the elevated expression levels of osteogenesis-related proteins (RUNX2, ALP, and OCN). Moreover, ALP staining showed that osteoblasts exposed to TiPs exhibited diminished ALP activity and impaired osteogenic differentiation, whereas KAE effectively ameliorated these alterations (Fig. 5*C*). ARS staining revealed that TiPs-exposed osteoblasts exhibited compromised mineralization capacity, while KAE partially enhanced the levels of extracellular matrix mineralization (Fig. 5*D*).

### KAE treatment promoted IRE1α/XBP1s activation and partially repressing IRE1α-mediated RIDD and JNK activation

**Figure 6:**
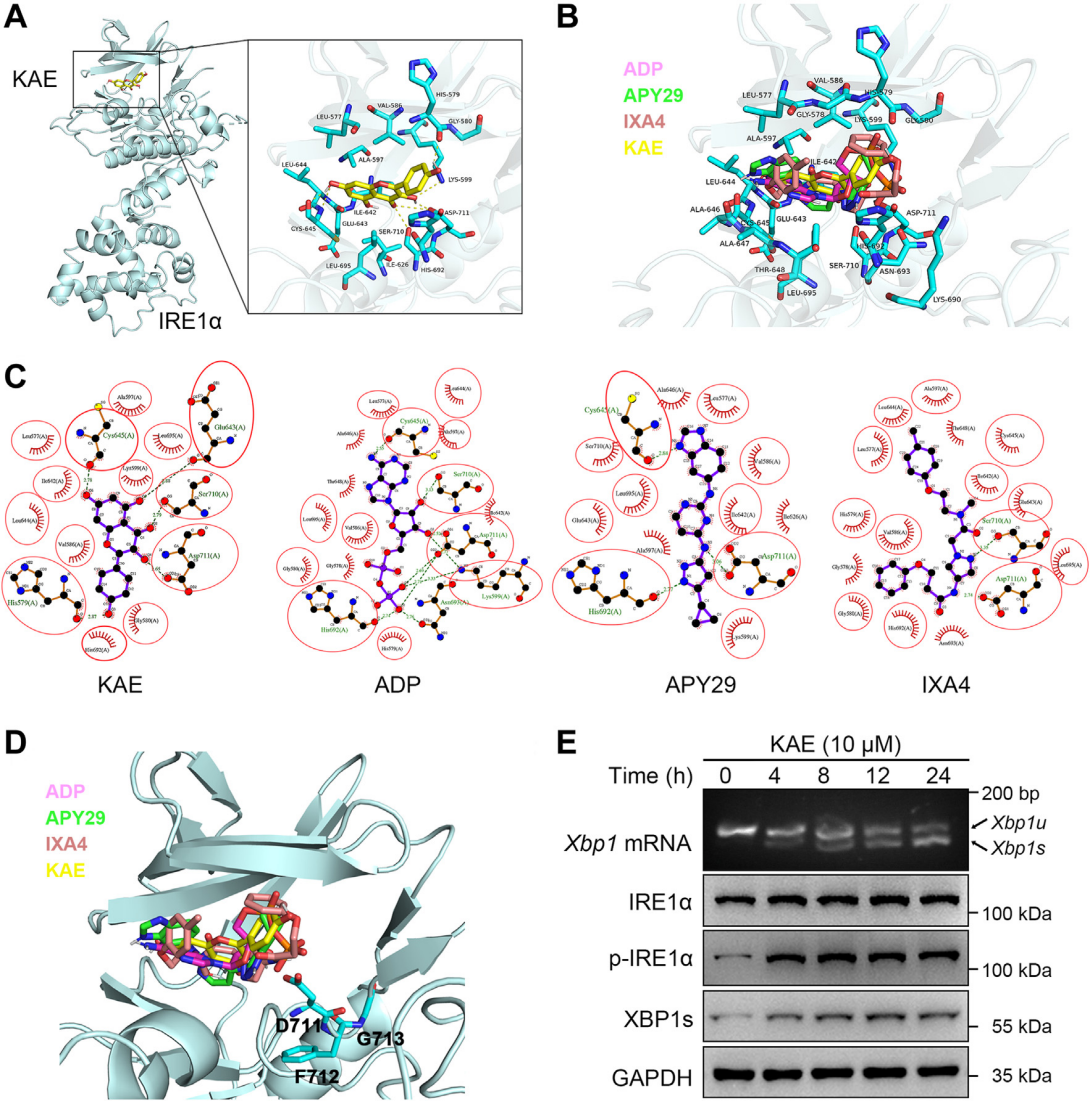
**KAE could enhance IRE1α activation to promote XBP1 mRNA splicing.***A* and *B*, molecular docking poses of KAE, ADP, APY29, and IXA4 within the nucleotide-binding site of the IRE1α kinase domain generated using AutoDock Suite 4.0. A superimposed representation was constructed using PyMOL, with *dashed yellow* lines representing hydrogen bonding interactions. *C*, LigPlot^+^ analysis (two-dimensional depiction) of the docked complex of KAE, ADP, APY29, and IXA4. *Red* circles highlight amino acid residues commonly interacting with all four compounds at the nucleotide-binding site. *Dashed green* lines indicate hydrogen bonding interactions between the amino acid residue and the compound. *D*, molecular docking poses of KAE, ADP, APY29, and IXA4 within the nucleotide-binding site with DFG-in confirmation (D711, F712, and G713) in the IRE1α kinase domain. *E*, agarose gel electrophoresis of XBP1 PCR products and Western blots of IRE1α, p- IRE1α, and XBP1s in osteoblast following treatment with KAE alone for 0, 4, 8, 12, and 24 h, respectively. KAE, Kaempferol.

The above-described findings suggested that KAE might attenuate osteoblast apoptosis and osteogenic reduction by suppressing TiPs-induced ER stress. The IRE1α–XBP1s pathway of UPR is known to play a pivotal role in maintaining ER proteostasis and cell survival under ER stress conditions (17, 20). Flavonol compounds, exemplified by quercetin, have shown promise in activating IRE1α RNase activity and thereby enhancing the cleavage of XBP1 mRNA (42). Given that KAE belongs to the flavonol class, it is reasonable to speculate that it shares similarities with quercetin in modulating ER stress responses. To explore this further, we conducted computational docking studies using Autodock to investigate the potential binding of KAE to the IRE1α molecule. Blind docking was conducted by encompassing the entire surface of the protein molecule. The result revealed an evident binding affinity of KAE towards the kinase pocket of IRE1α (Fig. 6*A*). Further analysis of the docked poses identified amino acid residues involved in the binding of KAE to the kinase pocket, exhibiting similarities to the interactions observed with ADP, APY29, and IXA4, which are recognized IRE1α RNase activators, suggesting the potential of KAE to activate IRE1α RNase activity (Fig. 6, *B*, *C*, and S5, *A*–*D*) (17, 43, 46, 47). Moreover, it is crucial to note that the confirmation of the DFG (Aspartic acid-phenylalanine-glycine) motif within the kinase domain of IRE1α, implicated in its RNase activation, ideally exhibits the DFG-in conformation rather than DFG-out (47). Analysis of the docked poses of IRE1α bound to ADP, APY29, IXA4, and KAE revealed a favorable conformation of the DFG-in motif, further supporting the potential of KAE to modulate IRE1α RNase activity (Fig. 6*D*).

To validate the findings from our docking analysis, we investigated the effects of KAE on the IRE1α–XBP1s pathway in osteoblasts *in vitro*. As shown in Figures 6*E* and S5*E*, KAE treatment promoted IRE1α activation and the splicing of XBP1 mRNA, along with increased protein expression levels of XBP1s, providing experimental evidence of KAE’s potential to activate IRE1α/XBP1s signaling in osteoblasts. Besides XBP1 splicing, IRE1α is recognized for its involvement in mediating RIDD of specific mRNA substrates and activating the JNK pathway (11, 16). Consequently, we further assessed the influence of KAE alone on IRE1α-mediated RIDD and JNK signaling in osteoblasts *in vitro*. Intriguingly, our results revealed that KAE did not exert any obvious impact on the mRNA expression of RIDD-specific substrates (*Bloc1s1*, *Scara3*, *Hgsnat*, *Col6α1*, *Pmp22*) and did not induce JNK phosphorylation (Fig. S5, *F* and *G*).

**Figure 7:**
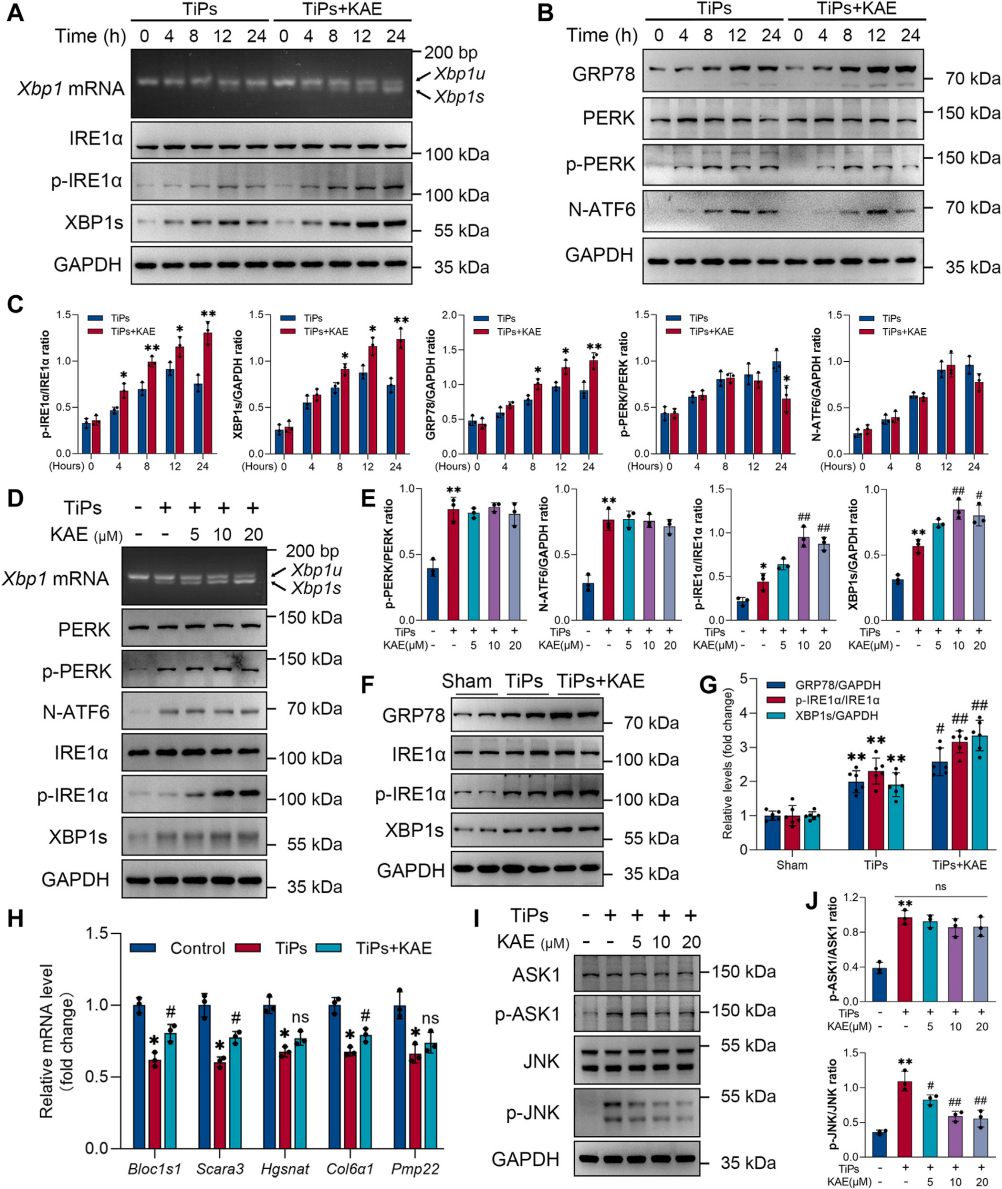
**KAE treatment promoted IRE1α/XBP1s activation while partially inhibiting IRE1α-mediated RIDD and JNK activation.***A*, agarose gel electrophoresis of XBP1 PCR products and Western blots of IRE1α, p-IRE1α, and XBP1s in osteoblasts following treatment with TiPs or TiPs + KAE for 0, 4, 8, 12, and 24 h, respectively. *B*, Western blots of GRP78, PERK, p-PERK, and N-ATF6 in osteoblasts following treatment with TiPs or TiPs + KAE for 0, 4, 8, 12, and 24 h, respectively. *C*, quantification of the Western blot results of (*A*) and (*B*), *n* = 3. *D* and *E*, agarose gel electrophoresis of XBP1 PCR products and Western blot analysis of PERK, p-PERK, N-ATF6, IRE1α, p-IRE1α, and XBP1s in osteoblasts following treatment for 12 h with different interventions. *n* = 3. *F* and *G*, Western blot analysis of GRP78, IRE1α, p-IRE1α, and XBP1s in mouse calvarial bone tissue samples per group. *n* = 6. *H*, qRT-PCR analysis of RIDD substrate (*Bloc1s1*, *Scara3*, *Hgsnat*, *Col6α1*, *Pmp22*) mRNA levels in osteoblasts following treatment with different interventions. *n* = 3. *I* and *J*, Western blot analysis of ASK1, p-ASK1, JNK, and p-JNK in osteoblasts following treatment for 24 h with different interventions. *n* = 3. All values are presented as mean ± SD. One-way ANOVA. ∗*p* < 0.05 and ∗∗*p* < 0.01 *versus* the control group or the sham group. ^#^*p* < 0.05 and ^##^*p* < 0.01 *versus* the TiPs group. *ns*, not statistically significant *versus* the TiPs group. JNK, c-Jun N-terminal kinase; KAE, Kaempferol; qRT-PCR, quantitative reverse transcription-PCR; RIDD, regulated IRE1α-dependent decay; TiPs, TiAl_6_V_4_ particles.

To probe the mechanism by which KAE regulates TiPs-induced ER stress, we further studied the effects of KAE on the activation of three signaling arms of the UPR triggered by ER stress. As shown in Figure 7, *A*–*C*, all branches of UPR were activated in osteoblasts upon initial exposure to TiPs. However, with prolonged exposure, signaling *via* IRE1α began to diminish, as evidenced by a decrease in IRE1α phosphorylation, XBP1 splicing, and XBP1s expression, while PERK and ATF6 signaling remained sustained. Remarkably, KAE treatment significantly enhanced IRE1α activation in osteoblasts exposed to TiPs, accompanied by an increase in XBP1 splicing and the expression of XBP1s and ER chaperone GRP78 (Fig. 7, *A*–*C*). With the reinforcement of IRE1α/XBP1s activation by KAE, there was a subsequent decrease in PERK phosphorylation and N-ATF6 expression during the later stages of TiPs exposure, likely due to alleviation of ER stress in osteoblasts (Fig. 7, *B* and *C*). However, it is noteworthy that during the early stages, KAE seemed to have no significant impact on PERK and ATF6 signaling in osteoblasts exposed to TiPs. To delve deeper into this observation, we examined the effects of various concentrations of KAE on the UPR branches in osteoblasts following 12 h of TiPs exposure. As shown in Figure 7, *D* and *E*, increasing concentrations of KAE promoted IRE1α/XBP1s activation while exerting no significant effects on PERK and ATF6 signaling. In addition, *in vivo* Western blot analysis revealed that KAE treatment also promoted IRE1α/XBP1s activation in the mouse models of TiPs-induced calvarial osteolysis (Fig. 7, *F* and *G*). These findings, combined with previous literature reports, suggest that KAE might primarily exert its anti-ER stress effects *via* the IRE1α–XBP1s pathway (11, 13, 20, 42, 43).

The modulatory mechanism of IRE1α during ER stress is complex and diverse. In addition to the splicing and activation of XBP1s, IRE1α can also modulate ER stress levels and cell fate by regulating RIDD and JNK signaling pathway (11, 44). Sustained ER stress triggers IRE1α to engage its RNase activity towards ER-resident mRNAs and certain anti-apoptotic miRNAs, an event termed RIDD, recognized as a mechanism contributing to ER stress-induced apoptosis (18, 44). Thus, we further investigated the effects of KAE on RIDD activity in osteoblasts exposed to TiPs. As shown in Figure 7*H*, TiPs exposure notably decreased the mRNA expression of RIDD-specific substrates, whereas KAE treatment partially mitigated these effects. In addition, upon prolonged ER stress, IRE1α can recruit TRAF2 and trigger ASK1/JNK activation, which in turn facilitates apoptotic processes (11, 16, 18). To examine the effects of KAE on IRE1α-mediated ASK1/JNK activation, we evaluated the phosphorylation levels of ASK1 and JNK in osteoblasts using Western blotting. The results revealed a significant increase in the phosphorylation level of ASK1 and JNK upon TiPs exposure (Fig. 7, *I* and *J*). However, KAE treatment notably reduced the level of JNK phosphorylation without affecting ASK1 phosphorylation, suggesting the potential of KAE to inhibit JNK activation.

### IRE1α–XBP1s pathway mediated the protective effects of KAE on osteoblasts exposed to TiPs

**Figure 8:**
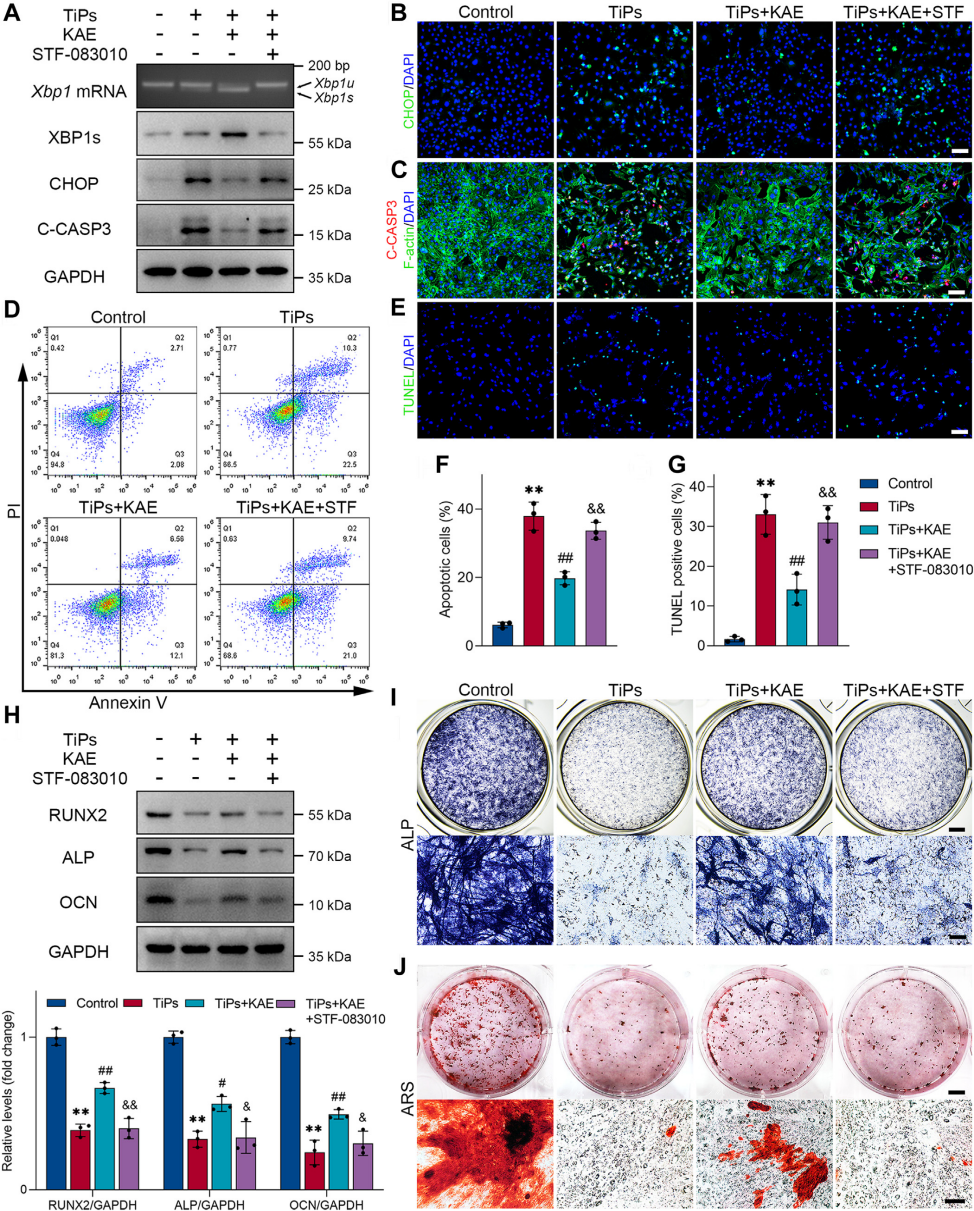
**IRE1α/XBP1s pathway mediated the protective effects of KAE on osteoblasts exposed to TiPs *in vitro*.***A*, agarose gel electrophoresis of XBP1 PCR products and Western blots of XBP1s, CHOP, and C-CASP3 in osteoblasts following treatment for 24 h with or without TiPs, KAE, and STF-083010 (50 μM). *B*, representative images of immunofluorescence staining for CHOP in each group. Scale bar represents 100 μm. *C*, representative images of immunofluorescence staining; *red* (C-CASP3), *green* (F-actin), *blue* (nuclei). Scale bar represents 100 μm. *D*, flow cytometry analysis of apoptosis using Annexin V/PI staining. *E*, representative images of TUNEL staining in each group. Scale bar represents 100 μm. *F*, cell apoptosis rate determined by flow cytometry analysis. *n* = 3. *G*, the percentages of TUNEL-positive cells in each group were quantified. *n* = 3. *H*, Western blot analysis of osteogenic markers (RUNX2, ALP, and OCN) in osteoblasts treated with different interventions after 3 days of osteogenic induction. *n* = 3. *I*, representative images of ALP staining for osteoblasts in each group. Scale bar represents 2 mm (top), 100 μm (*bottom*). *J*, representative images of ARS staining for osteoblasts in each group. Scale bar represents 5 mm (*top*), 250 μm (*bottom*). All values are presented as mean ± SD. One-way ANOVA. ∗∗*p* < 0.01 *versus* the control group. ^#^*p* < 0.05 and ^##^*p* < 0.01 *versus* the TiPs group. ^&^*p* < 0.05 and ^&&^*p* < 0.01 *versus* the TiPs + KAE group. ALP, alkaline phosphatase; ARS, Alizarin red S; KAE, Kaempferol; OCN, osteocalcin; RUNX2, Runt-related transcription factor 2; TiPs, TiAl_6_V_4_ particles; TUNEL, TdT-mediated dUTP nick end-labeling.

To validate the involvement of the IRE1α–XBP1s pathway in the protective effects of KAE on stressed osteoblasts during wear particle–induced osteolysis, we performed *in vitro* rescue experiments using STF-083010, a specific inhibitor of IRE1α endonuclease activity that does not affect its kinase activity, aiming to reduce the production of XBP1s (16, 43, 48, 49). PCR and Western blot analysis revealed that KAE treatment increased the splicing of XBP1 mRNA and the expression of XBP1s while decreasing the expression of CHOP and C-CASP3 in osteoblasts exposed to TiPs. Conversely, cotreatment with STF-083010 significantly blunted these changes in expression (Figs. 8*A* and S6, *A*–*C*). The results from immunofluorescence staining concurred with these findings (Fig. 8, *B* and *C*). Moreover, TUNEL staining results and flow cytometry data demonstrated that the elevation of the apoptotic rate induced by TiPs was significantly ameliorated by KAE treatment. However, cotreatment with STF-083010 abolished the KAE-mediated anti-apoptotic effects (Fig. 8, *D*–*G*). In addition, the results from osteogenic differentiation and mineralization experiments indicated that the favorable effects of KAE on osteogenesis were attenuated in the presence of STF-083010 (Fig. 8, *H*–*J*), further suggesting that the IRE1α–XBP1s pathway plays a critical role in mediating the protective effects of KAE on osteoblasts exposed to TiPs.

**Figure 9:**
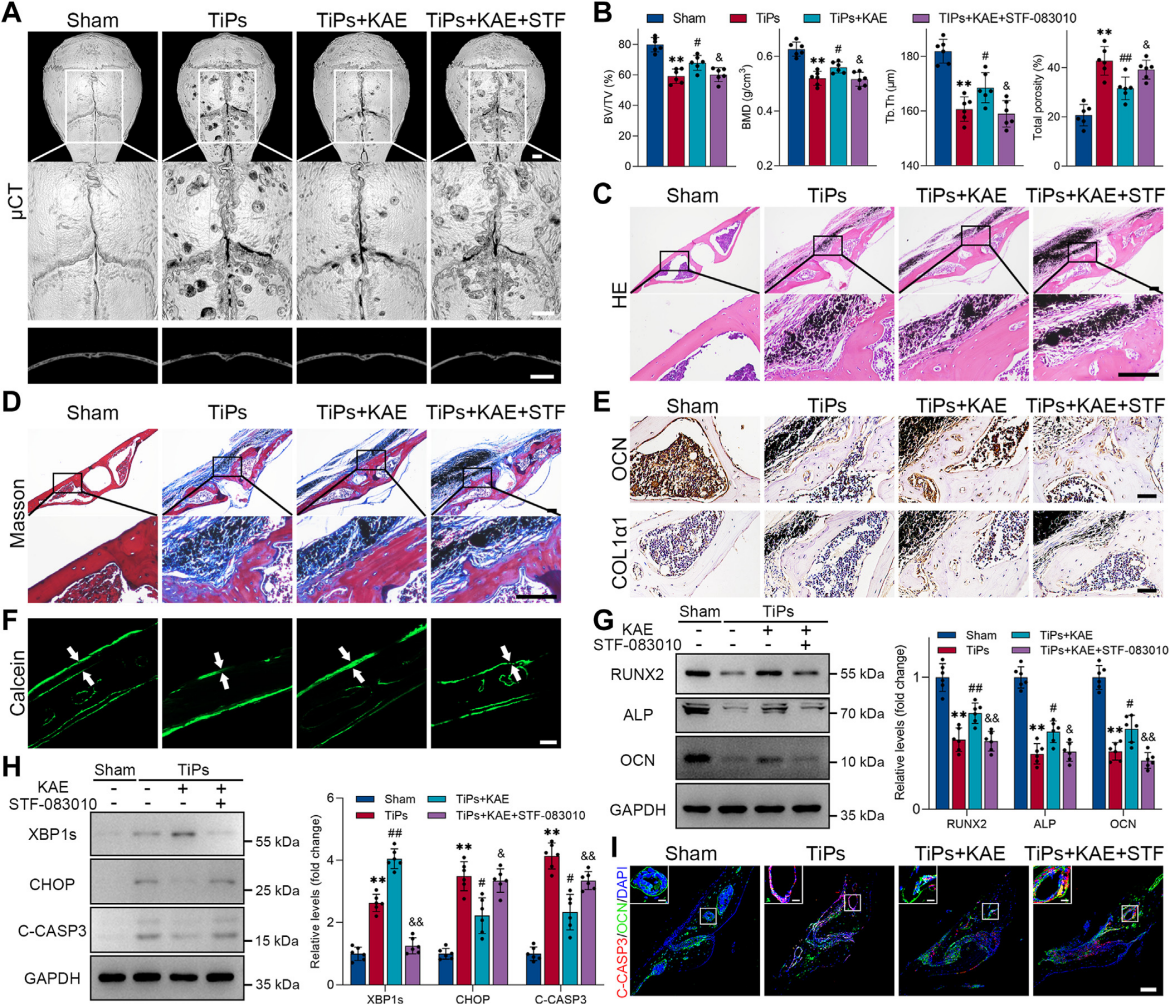
**IRE1α/XBP1s pathway mediated the protective effects of KAE on stressed osteoblasts in the mouse model of TiPs-induced osteolysis.***A*, representative micro-CT (μCT) three-dimensional reconstructed images (*top* and *middle*) and cross-sectional images (*bottom*) of mouse calvaria in each group. Scale bar represents 1 mm. *B*, quantitative analysis of bone morphometric parameters, including BV/TV (%), BMD (g/cm^3^), Tb.Th (μm), and total porosity (%), *n* = 6. *C*, representative H&E staining images of mouse calvarial sections from each group. Scale bar represents 100 μm. *D*, representative Masson’s trichrome staining of mouse calvarial sections from each group. Scale bar represents 100 μm. *E*, representative images of immunohistochemical staining for OCN (*upper* images) and COL1α1 (*lower* images). Scale bar represents 50 μm. *F*, representative images of calcein double labeling with a 10-days interval. Scale bar represents 50 μm. *G*, Western blot analysis of osteogenic markers (RUNX2, ALP, OCN) in mouse calvarial bone tissue samples per group. *n* = 6. *H*, Western blot analysis of XBP1s, CHOP, and C-CASP3 in mouse calvarial bone tissue samples per group. *n* = 6. *I*, representative images of immunofluorescence double staining for C-CASP3 (*red*) and OCN (*green*) in histological sections of the mouse calvaria per group. Scale bar represents 100 μm; scale bar (enlarged) represents 20 μm. All values are presented as mean ± SD. One-way ANOVA. ∗∗*p* < 0.01 *versus* the sham group. ^#^*p* < 0.05 and ^##^*p* < 0.01 *versus* the TiPs group. ^&^*p* < 0.05 and ^&&^*p* < 0.01 *versus* the TiPs + KAE group. ALP, alkaline phosphatase; BMD, bone mineral density; BV/TV, bone volume to total volume; KAE, Kaempferol; OCN, osteocalcin; micro-CT, micro-computed tomography; RUNX2, Runt-related transcription factor 2; Tb.Th, total porosity and trabecular thickness; TiPs, TiAl_6_V_4_ particles.

To further corroborate the *in vitro* findings, we subsequently investigated the impact of STF-083010 on the protective effect of KAE on stressed osteoblasts in *in vivo* models. The results obtained from micro-CT analysis and H&E staining revealed that TiPs-induced calvarial osteolysis was markedly alleviated by KAE treatment, whereas co-administration of STF-083010 counteracted the bone-sparing effects mediated by KAE (Figs. 9, *A*–*C* and S7, *A* and *B*). Furthermore, we evaluated the effects of STF-083010 on bone formation in mouse calvaria and observed that co-administration of STF-083010 largely attenuated the promoting role of KAE in osteogenesis *in vivo* (Figs. 9, *D*–*G* and S7, *C*–*E*). Western blot analysis indicated that KAE treatment increased the expression of XBP1s and decreased the expression of CHOP and C-CASP3 *in vivo*, whereas cotreatment with STF-083010 blunted these alterations in expression (Fig. 9*H*). Consistently, as shown in Figure 9*I*, the results of immunofluorescence staining demonstrated that KAE treatment effectively mitigated TiPs-induced osteoblast apoptosis in mouse calvaria, whereas cotreatment with STF-083010 counteracted the anti-apoptotic effects mediated by KAE, providing further evidence that the IRE1α–XBP1s pathway mediates the protective effects of KAE on TiPs-induced osteoblast apoptosis *in vivo*.

## Discussion

**Figure 10:**
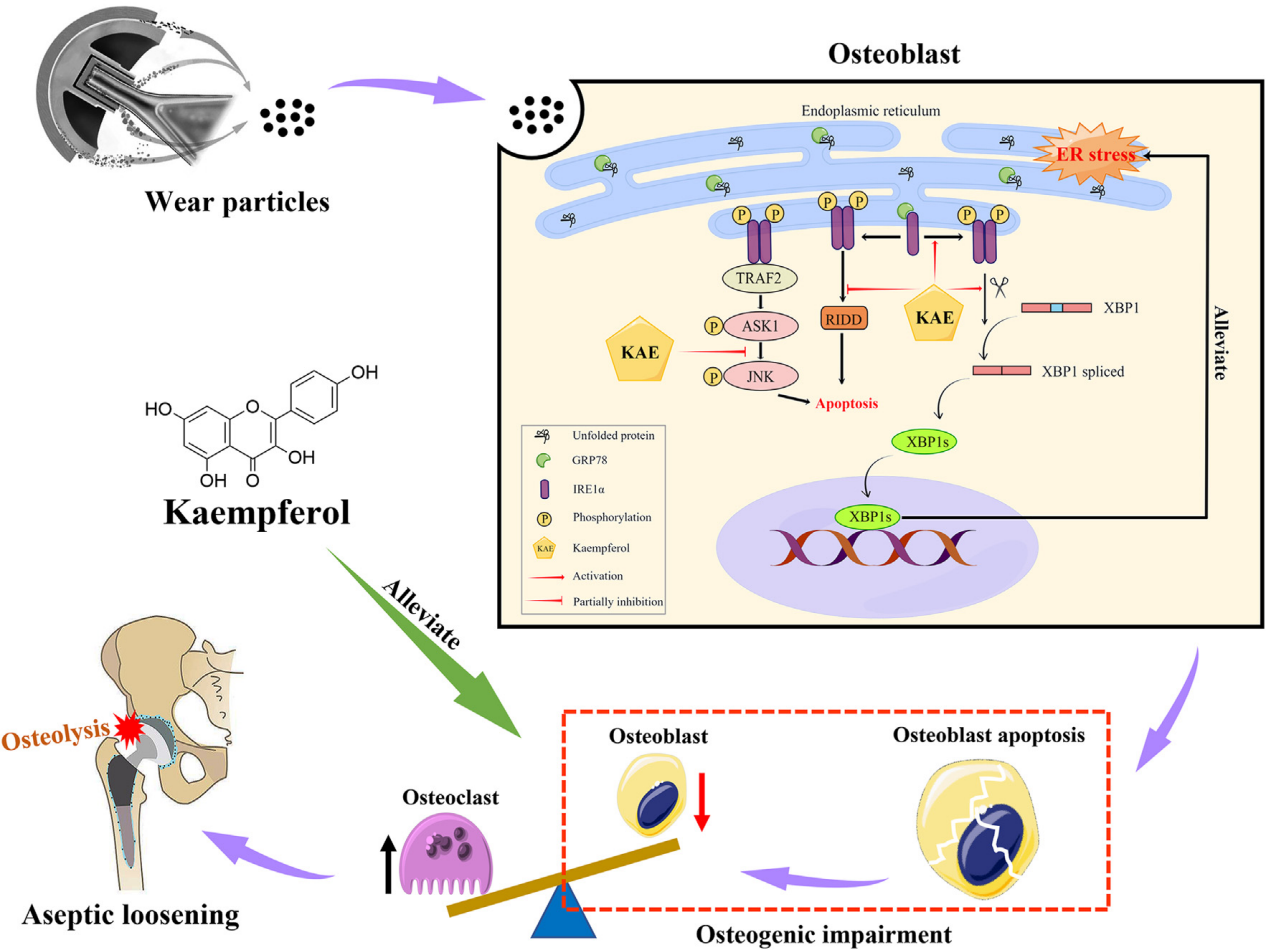
**Schematic representation of a possible mechanism underlying how KAE alleviated ER stress-mediated osteogenic impairment in osteoblasts exposed to wear particles.** ER, endoplasmic reticulum; KAE, Kaempferol.

In this study, we observed that KAE could effectively ameliorate particle-induced osteolysis by mitigating the impairment of osteogenesis. Furthermore, we demonstrated that KAE could attenuate ER stress-mediated apoptosis in osteoblasts exposed to particles, both *in vitro* and *in vivo*. Mechanistically, as summarized in Figure 10, our findings indicated that KAE mitigated ER stress-mediated apoptosis by upregulating the IRE1α–XBP1s pathway, while concurrently partially suppressing the activation of the IRE1α-regulated RIDD and JNK signaling, thereby maintaining osteoblast viability and functionality. Hence, our research underscores the promising clinical potential of KAE as a prospective therapeutic agent for the prevention and treatment of periprosthetic osteolysis. Additionally, our study highlights the IRE1α–XBP1s pathway as a potential therapeutic target for the prevention and treatment of periprosthetic osteolysis, providing novel insights for future drug development in combating aseptic loosening of artificial joints.

The pathogenesis of aseptic loosening is a complex biological process, with wear particle–induced osteolysis being its core mechanism (6, 50, 51). Wear particles have been demonstrated to result in decreased bone formation around the joint prosthesis, which is attributed to the detrimental effects of wear particles on the viability, proliferation, differentiation, and functionality of osteoblasts (4, 5, 6). However, previous research into strategies for preventing and treating periprosthetic osteolysis primarily emphasized suppressing wear particle–induced periprosthetic inflammation and excessive generation of osteoclast, with a relatively limited focus on intervening in osteoblast-mediated bone formation (5, 6, 7). As of now, nonsurgical pharmacotherapies approved by the Food and Drug administration to arrest aseptic loosening remain absent (34, 52). Consequently, it is necessary to search for potential therapeutic targets and agents for the prevention and treatment of wear particle–induced osteolysis. Our recent research has demonstrated that ER stress mediates wear particle–induced osteoblast apoptosis and osteogenic reduction, highlighting the potential of modulating ER stress in osteoblasts as a promising strategy to improve periprosthetic bone formation and prevent aseptic loosening (7).

As the most evolutionarily conserved sensor of ER stress, IRE1α plays a central role in maintaining ER homeostasis (11, 14). Previous studies have suggested that upon prolonged ER stress, IRE1α signaling becomes attenuated, contrasting with the sustained activation of PERK signaling, which mediates CHOP-induced apoptosis (13, 20, 53). This attenuation of IRE1α signaling has been proposed as a potential mechanism underlying the transition from the adaptive to the pro-apoptotic phase of the UPR. Aligned with this notion, our observations revealed a weakening of IRE1α/XBP1s activation during the later stages of TiPs exposure, possibly compromising the pro-survival outcomes associated with XBP1s expression. In this study, we demonstrated that KAE, a natural flavonol compound, exerts a specific effect in promoting IRE1α activation and enhancing its RNase activity, leading to an increase in the splicing of XBP1 mRNA and the expression of XBP1s. XBP1s, in turn, upregulates the expression of crucial genes involved in restoring ER function, such as GRP78, assisting cells in addressing and adapting to ER stress, thus maintaining ER homeostasis (13, 14, 18). As anticipated, we noted an increase in GRP78 levels *in vitro* and *in vivo*, likely attributed to *de novo* synthesis resulting from the enhanced activation of the IRE1α–XBP1s pathway induced by KAE.

Moreover, IRE1α RNase activation also promotes the degradation of mRNA localized to the ER, a process known as RIDD (16, 18). Research has shown that activated IRE1α RNase employs distinct mechanisms for cleaving XBP1 mRNA *versus* RIDD substrates (54, 55). Specifically, a catalytically active IRE1α unit engaged in XBP1 mRNA splicing emerges within the IRE1α oligomer, while IRE1α involved in RIDD resides within an IRE1 monomer/dimer configuration. However, our findings showed that when KAE was applied solely to osteoblasts, it did not exert any discernible impact on the mRNA expression of RIDD-specific substrates. This observation suggests a potential association with conformational changes in IRE1α, which warrants further exploration in future studies to fully elucidate the underlying mechanisms. Notably, previous studies have indicated that when IRE1α is allosterically activated by modulation with inhibitors targeting the kinase domain, generalized mRNA degradation and apoptosis were not observed, despite intact XBP1 splicing (11, 56). Our molecular docking analysis results revealed that the mode of action of KAE in activating IRE1α RNase activity appears analogous to that of a type Ⅰ kinase inhibitor, such as APY29, which induces allosteric activation of the IRE1α RNase through the remodeling of the kinase pocket by ATP-competitive ligands (43, 47, 57, 58). Furthermore, our investigation revealed that KAE shares a similar binding site on IRE1α with the recently reported selective IRE1α/XBP1s activator, IXA4 (16, 17). Although the specific mechanism of action for IXA4 remains to be fully elucidated, it is noteworthy that IXA4 has been reported to selectively activate IRE1α RNase activity to splice XBP1 mRNA and promote XBP1s expression, without inducing RIDD (16, 17, 58, 59). These findings provide valuable insights into the mechanism by which KAE modulates IRE1α RNase activity and highlight its potential as a pharmacological agent for modulating ER stress response pathways. Additionally, RIDD is typically induced under conditions of sustained high-level IRE1α activation during prolonged ER stress (16). This may be attributed to the transient and moderate activation of IRE1α induced by KAE, which might not be sufficient to trigger RIDD activity. Further studies are warranted in the future to delineate the precise mechanism of action for KAE, as well as its potency in other experimental models and under varying conditions. Moreover, we discovered that TiPs exposure notably decreased the mRNA expression of RIDD-specific substrates, whereas KAE treatment partially mitigated these effects in osteoblasts exposed to particles. This could be attributed to KAE’s timely alleviating of the ER stress induced by TiPs exposure.

It is noteworthy that prolonged activation of IRE1α can initiate the activation of the ASK1–JNK pathway, culminating in cell apoptosis and autophagy (11). Additionally, a previous study conducted by our group confirmed that IRE1α/JNK-mediated autophagy is a crucial mechanism in wear particle–induced osteoblast apoptosis (3). In the present study, we were surprised to observe that KAE significantly inhibited the phosphorylation of JNK in osteoblasts exposed to particles, while showing no obvious impact on ASK1 phosphorylation. This suggests that KAE might act as an inhibitor of JNK activation, possibly by modulating upstream signaling events. Moreover, in our recent work, we found that KAE also inhibited RANKL-induced JNK phosphorylation during osteoclast differentiation (34). Similar findings have been reported in other studies, further supporting the potential of KAE as a JNK pathway modulator (60, 61, 62). These compelling findings shed light on the capacity of KAE to inhibit JNK activation, marking it as a promising candidate for further exploration and development as a JNK-targeting therapeutic agent.

In recent years, KAE has garnered attention for its potential in modulatory effects on ER stress across a variety of disease models. Wang *et al.* demonstrated that KAE could mitigate D-GalN/LPS–induced acute liver failure by regulating ER stress through upregulation of GRP78 and inhibition of CHOP (35). Kim *et al.* observed that KAE alleviated ER stress-mediated inflammation and insulin resistance in HepG2 cells treated with thapsigargin (36). Park et al. found that KAE alleviated ER stress in airway epithelial cells by inhibiting the IRE1α–TRAF2–JNK activation, thereby suppressing asthmatic mucus hypersecretion and goblet cell hyperplasia (37). KAE was also reported to exhibit protective effects on ischemia/reperfusion-induced cardiac damage through inhibition of ER stress (40). Our present study provided evidence that KAE could mitigate particle-induced reduction in bone formation by inhibiting ER stress-mediated osteoblast apoptosis and osteogenic impairment. Furthermore, we shed light on the potential mechanism by which KAE alleviates ER stress in osteoblasts exposed to particles, underscoring the involvement of the IRE1α–XBP1s pathway in mediating KAE’s protective effects.

Increasing IRE1α/XBP1s activation has been established to ameliorate diverse diseases associated with ER stress (16, 17, 18). For instance, XBP1s overexpression has shown neuroprotective effects in multiple animal models of neurodegenerative diseases, such as Parkinson’s disease, Huntington’s disease, and peripheral nerve injury (17). IRE1α/XBP1s activation has been reported to alleviate ER stress induced by myocardial ischemia, effectively inhibiting cardiomyocyte apoptosis and reducing the infarct area in a myocardial infarction model (18, 63). A study by Jiang *et al.* demonstrated that activating the IRE1α–XBP1s pathway could mitigate ER stress-mediated apoptosis in intestinal epithelial cells, thus alleviating intestinal injury (64). Moreover, IRE1α/XBP1s activation has shown benefits in models of other disorders, including obesity and diabetes (16, 65). Our findings suggested that the IRE1α–XBP1s pathway may also serve as a potential therapeutic target for preventing and treating aseptic loosening. Additionally, the IRE1α–XBP1s pathway plays a pivotal regulatory role during osteogenic differentiation through the modulation of the expression of osteogenesis-related genes (21, 22, 23). Given that our study revealed KAE as a potential activator of the IRE1α–XBP1s pathway, it is conceivable that this pathway may also be involved in mediating the promotion effects of KAE on osteoblast ability in other models, as well as its osteoprotective effects against other inducers like glucocorticoids (32, 66, 67).

Currently, the Food and Drug administration has not approved any nonsurgical pharmacotherapies for halting the progression of aseptic loosening (52). This deficiency has driven researchers to actively search for potential agents capable of preventing and treating aseptic loosening. In recent years, alternative natural compounds have garnered considerable attention in managing osteolytic diseases, attributed to their favorable biosafety, potent bioactivity, and synergistic multi-target characteristics (68, 69). KAE has been recognized as a central active constituent in various traditional Chinese herbal medicines known for their osteoprotective properties, such as *Kaempferia galanga* L., *Eucommia ulmoides* Oliv., and *Cuscuta chinensis* Lam (70, 71, 72). Current evidence indicates that KAE may positively influence bone metabolism and address the imbalance between bone formation and bone resorption at the cellular level by enhancing osteoblast activity while concurrently inhibiting osteoclast formation (32). Our previous research has substantiated KAE’s ability to suppress particle-induced osteoclast activation and inflammatory responses in a mouse calvarial osteolysis model (34). The current study has further established that KAE can also alleviate particle-induced apoptosis in osteoblasts and reduce osteogenic activity *in vivo*. These findings reinforce the dual regulatory role of KAE, highlighting its potential to ameliorate particle-induced osteolysis by modulating both osteogenic and osteoclastic processes. Moreover, while various mechanisms have been reported regarding the regulation of osteoblast activity by KAE, such as through mTOR signaling, estrogen receptor signaling, and Wnt/β-catenin signaling (31, 32, 33), our study is pioneering in demonstrating that KAE maintains osteoblast activity by modulating ER stress in the context of aseptic loosening. It is crucial to acknowledge that the modulation of ER stress represents merely one aspect of KAE’s multifaceted therapeutic actions. Further investigation is imperative in future studies to elucidate the precise mechanisms underlying KAE’s therapeutic potential, including its effects on various downstream signaling pathways.

There are certain limitations that should be noted in our study. Firstly, our research primarily focused on exploring the impact of KAE on bone formation in a mouse calvarial osteolysis model and evaluating its effects on osteoblast viability and functionality in *in vitro* models. However, a more extensive evaluation of KAE’s effects on diverse tissues and cell types is warranted in future study to provide a comprehensive understanding of its potential applications in periprosthetic osteolysis. Secondly, although we have demonstrated that KAE mitigates ER stress-mediated apoptosis in osteoblasts exposed to wear particles by promoting IRE1α/XBP1s activation, the specific downstream mechanisms remain to be elucidated. Thirdly, this study exclusively utilized metal wear particles in experiments, but we did not extensively investigate other types of implant wear particles, such as polyethylene and ceramic particles. In future research, we intend to broaden our investigation by incorporating diverse wear particle types to validate and extend our findings comprehensively.

## Experimental procedures

### Particle preparation and characterization

TiPs were acquired from the prosthesis of patients who underwent revision hip arthroplasty due to aseptic loosening (34). Following a protocol previously described by our group, the prosthesis underwent sterilization and was subsequently transformed into nanoscale wear particles using a fabricated high-vacuum three-electrode direct current system under the following conditions: a vacuum pressure of 10^−3^ Pa, a gas mixture of 0.04 MPa argon and hydrogen in a 3:2 (v/v) ratio, and a cathode current of 650 A (34, 73). All clinical procedures were granted ethical approval by the Clinical Ethics Committee of the Nanjing Jinling Hospital, the Affiliated Hospital of Nanjing University Medical School (license no. 2019NZGKL-012), and conformed to the principles of the Helsinki Declaration. Informed consent was obtained from all the participants in this study.

The obtained TiPs were subjected to characterization using scanning electron microscopy (Regulus-8100) and transmission electron microscopy (HT7800) (Fig. S1*A*). As previously described by our research group, the average particle diameter of the TiPs was 51.7 nm (7, 34, 74). Previous reports have indicated that metal wear particles retrieved from periprosthetic tissues of patients with aseptic loosening exhibit a particle size at the nanometer scale (34, 75). Thus, the TiPs prepared in this study are well-suited for simulating the wear particles generated by metallic implants within the human body. Endotoxin removal procedures were executed as previously reported (76), resulting in endotoxin levels for TiPs of less than 0.25% EU/ml, as determined using the Pierce LAL Chromogenic Endotoxin Quantitation Kit (Thermo Fisher Scientific, 88282). The resulting particles were suspended in sterile PBS (PBS; BOSTER, AR0030) at a 50 mg/ml stock concentration and then autoclaved for 15 min at 121 °C for sterilization. Before use, the TiPs suspension was ultrasonicated for 20 min.

### *In vivo* calvarial osteolysis mouse model

The mouse calvarial osteolysis model was performed as previously described by our research group (34, 73, 77). The C57BL/6J mice (male, 8–10 weeks old, 22–25 g) used in our study were purchased from the Model Animal Research Center of Nanjing University. All mice were housed in a conventional clean room under constant temperature (22–25 °C), relative humidity (55–60%), and 12 h light-dark cycle conditions with unlimited access to food and water throughout all experiment periods. Mice were randomly divided into four groups (12 mice per group): (1) group Ⅰ, Sham-operated mice; (2) group Ⅱ, TiPs-treated mice; (3) group Ⅲ, mice treated with TiPs receiving KAE; (4) group Ⅳ, mice treated with TiPs receiving KAE and STF-083010.

Briefly, mice were anesthetized with intraperitoneal phenobarbitone (40 mg/kg, 1%), and the skin and periosteum were separated to expose the skull. Forty microliters of 50 mg/ml TiPs suspension were placed under the periosteum at the middle suture of the exposed calvaria (3, 34). The sham-operated mice underwent the same operation without TiPs treatment. KAE (MCE, HY-14590-22807) (Fig. S1*B*) was dissolved in PBS with 0.5% sodium carboxymethyl cellulose (CMC-Na), and mice in group Ⅲ and Ⅳ were administered orally once daily after surgery at a dose of 5 mg/kg KAE by gastric gavage (32, 34). For rescue experiments, mice in group Ⅳ received a once-daily intraperitoneal injection of STF-083010 (5 mg/kg, Selleck, S7771) after surgery (16). Two weeks following treatment, all mice were sacrificed, and the calvariae and major organs were harvested for further analysis. Additionally, the serum samples from each group were collected for biochemical index detection. All animal protocols for the experiments were approved by the Animal Ethics Committee of the Nanjing Jinling Hospital, the Affiliated Hospital of Nanjing University Medical School (license no. 2019JLHGKJDWLS-073), per the National Institutes of Health Guide for the Care and Use of Laboratory Animals.

### micro-CT scanning and analysis

All samples of mouse calvaria were subjected to high-resolution micro-CT scanning using a SkyScan 1176 scanner (Bruker). The scanning parameters included a voxel size of 18 μm, an X-ray source set at 50 kV, and a current of 455 μA. A square region of interest (ROI, 5 mm × 5 mm × 1 mm) around the sagittal suture was chosen for calculating the following bone morphometric parameters: bone volume-to-total volume, bone mineral density, total porosity and trabecular thickness (34, 77). CTvox and CTAn software (https://www.bruker.com/) provided by SkyScan were employed for three-dimensional reconstruction and image analysis (78).

### Histological and immunohistochemical staining

After micro-CT scanning and analysis, all samples of mouse calvaria and major organs were decalcified with 10% EDTA at 4 °C for 14 days and embedded in paraffin. Then, the tissue samples were sliced into 5 μm sections with an RM2235 microtome (Leica). H&E staining was performed using an H&E staining Kit (Leagene, DH0006) according to standard H&E staining procedures. For TRAP staining, a TRAP staining kit (Wako, 294–67001) was used to assess osteoclast activity following the manufacturer’s protocol. For Masson’s trichrome staining, a Masson’s trichrome staining kit (KeyGEN, KGMST-8004) was applied for collagen staining according to the manufacturer’s instructions

Immunohistochemical (IHC) staining was performed using an IHC kit (KeyGEN, KGOS300) as previously described (7, 34). The primary antibodies used for the IHC assay were as follows: anti-OCN (osteocalcin) rabbit IgG (Affinity, DF12303), anti-COL1α1 (Collagen Type Ⅰ Alpha 1) rabbit IgG (Boster, PB0981).

For calcein fluorescent double-labeling assay, mice were intraperitoneally injected with 20 mg/kg calcein (Sigma-Aldrich, C0875) at 12 and 2 days respectively before sacrifice (3, 7). After fixation, dehydration, and embedment, the calvarias of mice were cut into 10-μm-thick frozen coronal sections using a cryostat microtome (Leica CM1900). The calcein double-labeling interval was determined using an LSM 980 confocal microscope (Zeiss). The mineral apposition rate (μm/day) was calculated by dividing the mean interval distance by the inter-label time of 10 days.

### *In vivo* biosafety evaluation

To evaluate the *in vivo* biosafety of TiPs and KAE, blood samples were harvested *via* cardiac puncture, allowed to clot at room temperature, and then centrifuged at 3000 rpm for 15 min to collect serum. Serum biochemical parameters including liver function indicators (alanine aminotransferase, aspartate aminotransferase) and renal function indicators (blood urea nitrogen, creatine) were measured using an automatic blood biochemical analyzer (DRI-CHEM NX700i; Fujifilm). Furthermore, to evaluate the systemic toxicity *in vivo*, major organs (heart, liver, spleen, lung, and kidney) were collected for H&E staining and histological analysis.

### Western blotting

Total proteins from cells or bone specimens were extracted with a cell lysis buffer (Beyotime, P0013) containing protease and phosphatase inhibitor cocktail mix (Beyotime, P1045) as described previously, and protein concentration was determined using a BCA protein concentration detection kit (Solarbio, PC0020). Then, protein extracts were boiled for 5 min at 100 °C with 5×SDS loading buffer (Servicebio, G2075). Thirty micrograms of protein from each sample were separated on 12.5% SDS-PAGE gels (Epizyme, PG113) and transferred to polyvinylidene difluoride membrane (0.22 μm, Millipore, ISEQ00010). The transferred membranes were blocked with QuickBlock blocking buffer (Beyotime, P0252FT) for 15 min at room temperature to reduce nonspecific binding and subsequently incubated in primary antibody solution for 4 °C overnight. Next, the membranes were washed three times with 1×TBST and incubated with HRP-conjugated secondary antibody solution for 1 h at room temperature and then washed with 1×TBST. Finally, the membranes were exposed to a chemiluminescence (ECL) developing solution (Vazyme, E411-05) and analyzed using a Tanon 4200SF imaging analysis system (Tanon). Western blotting was performed using the following specific antibodies: anti-RUNX2 mouse IgG (Abcam, ab76956), anti-ALP rabbit IgG (Abcam, ab224335), anti-OCN rabbit IgG (Affinity, DF12303), anti-CHOP rabbit IgG (Affinity, AF6277), anti-C-CASP3 rabbit IgG (Cell Signaling Technology, 9664), anti-BAX rabbit IgG (Abcam, ab32503), anti-BCL2 rabbit IgG (Cell Signaling Technology, 3498), anti-Cytochrome c rabbit IgG (Abcam, ab133504), anti-GRP78 rabbit IgG (Abcam, ab21685), anti-PERK rabbit IgG (Affinity, AF5304), anti-phospho-PERK rabbit IgG (Affinity, DF7576), anti-ATF6 rabbit IgG (Abcam, ab37149), anti-IRE1α rabbit IgG (Abcam, ab37073), anti-phospho-IRE1α rabbit IgG (Abcam, ab48187), anti-XBP1s rabbit IgG (Cell Signaling Technology, 40435), anti-ASK1 rabbit IgG (Abcam, ab45178), anti-phospho-ASK1 rabbit IgG (Abcam, ab278547), anti-JNK rabbit IgG (Cell Signaling Technology, 9251), anti-phospho-JNK rabbit IgG (Cell Signaling Technology, 9258), anti-GAPDH rabbit IgG (Affinity, AF7021), Goat anti-rabbit IgG-HRP (Proteintech, PR30011), and Goat anti-mouse IgG-HRP (Proteintech, PR30012). Uncropped Western blot images are shown in Fig. S8.

### Isolation and culture of primary mouse osteoblasts

Primary mouse osteoblasts were derived from neonatal mouse (C57BL/6J, 1–3 days) calvaria as described previously (79, 80). Briefly, the calvariae were dissected aseptically, rinsed with sterile PBS, and cut into bone pieces using a sterile ophthalmic scissor. Subsequently, the bone pieces were subjected to sequential digestion in a 0.1% collagenase (Sigma-Aldrich, C6885) and 0.125% trypsin (Beyotime, C0207) mixture at 37 °C for 20 min. After digestion, the isolated cells were cultured in α-Minimal Essential Medium (Gibco, 12571063) containing 10% fetal bovine serum (Gibco, 10099141) and 1% Penicillin-Streptomycin (NCM Biotech, C100C5) at 37 °C incubator with 5% CO_2_ and used for further experiments when they reached passages 3 to 5. For osteoblast differentiation, primary mouse osteoblasts were reseeded and cultured in α-Minimal Essential Medium containing 10% fetal bovine serum, 10 nM dexamethasone (Beyotime, ST1254), 50 μg/ml ascorbic acid (Beyotime, ST1434), and 5 mM β-glycerophosphate (Sigma, G9422).

### Cell viability assay

Cell viability was examined using a CCK-8 assay kit (Dojindo, CK04) according to the manufacturer’s protocol. To evaluate the cytotoxic effects of KAE on primary osteoblasts at varying concentrations, cells were seeded in 96-well plates at a density of 8 × 10^3^ cells per well in triplicate. After overnight incubation, cells were exposed to various concentrations of KAE (0, 1, 5, 10, 20, and 40 μM) for 24, 48, and 72 h, respectively. To simulate an *in vitro* osteolytic microenvironment, TiPs were diluted in culture medium to achieve concentrations spanning from 10 to 200 μg/ml and then applied to primary osteoblasts (74, 81, 82). Subsequently, cell viability was assessed after incubation periods of 12, 24, and 48 h, respectively. To evaluate the effects of KAE on TiPs cytotoxicity, primary osteoblasts were pretreated with varying concentrations (0, 1, 5, 10, and 20 μM) of KAE for 6 h, followed by cotreatment with TiPs (50 μg/ml) for 0, 12, 24, and 48 h, respectively (74).

After treatment, 10 μl of CCK-8 solution was added to each well and incubation continued for 2 h at 37 °C. Finally, the absorbance was measured at 450 nm using a Varioskan LUX multimode microplate reader (Thermo Fisher Scientific).

### Flow cytometry analysis

Primary osteoblasts were seeded into 6-well plates at a density of 1 × 10^6^ cells per well and incubated with or without KAE for 6 h before being cotreated with TiPs for another 24 h. Subsequently, cells were collected and stained with Annexin V-FITC and propidium iodide using an Apoptosis Detection Kit (Solarbio, CA1020) following the manufacturer’s instructions and then analyzed on an Attune NxT flow cytometer (Thermo Fisher Scientific). The apoptosis levels were measured using FlowJo software (Version X 10.0.7, https://www.flowjo.com/) (3, 34).

### TUNEL staining

TUNEL staining was performed using the TUNEL Apoptosis Detection kit (Servicebio, G1501) following the manufacturer’s instructions. In brief, cells were fixed in 4% paraformaldehyde for 15 min and permeabilized with 0.1% Triton X-100 for 5 min. Subsequently, the cells were incubated with a staining solution containing TdT enzyme, FITC-dUTP, and equilibration buffer for 1 h at 37 °C and then stained with 4′,6-Diamidino-2-phenylindole (Solarbio, C0060). TUNEL-positive cells were observed and imaged using an LSM 980 confocal microscope (Zeiss), and cells exhibiting green fluorescence were considered as apoptotic cells.

### Immunofluorescence staining

To detect ER stress and apoptosis in osteoblasts, the fixed cells or calvarial bone sections were permeabilized with 0.1% Triton X-100 and blocked with 5% bovine serum albumin (Beyotime, ST023). Subsequently, the cells or sections were incubated with primary antibodies for CHOP (Affinity, AF6277), C-CASP3 (Cell Signaling Technology, 9664), or OCN (Santa Cruz Biotechnology, sc-390877) at 4 °C overnight (3, 83) and then incubated with appropriate secondary antibodies labeled with Alexa Fluor 488 or Alexa Fluor 546 (Invitrogen) for 1 h at room temperature. For F-actin cytoskeleton staining of osteoblast, the prepared cells were directly stained with Phalloidin-iFluor 488 (Abcam, ab176753) for 1 h at 37 °C, following the manufacturer’s instructions. Finally, cells and sections were viewed and photographed using a confocal fluorescence microscope (LSM 980, ZEISS) after staining nuclei with 4′,6-Diamidino-2-phenylindole (Solarbio, C0060).

### ALP staining

For ALP staining, primary mouse osteoblasts were fixed in 4% paraformaldehyde for 15 min following 7 days of osteogenic induction and then rinsed three times with PBS. Next, the cells were stained using a BCIP/NBT ALP Color Development Kit (Beyotime, C3206), according to the manufacturer’s instructions. Finally, the osteogenic differentiation level of osteoblasts was evaluated based on the intensity of ALP staining.

### ARS staining

After 14 days of osteogenic induction, primary mouse osteoblasts were fixed in 4% paraformaldehyde for 15 min and then rinsed with double-distilled H_2_O. Subsequently, the cells were stained using an ARS Staining Kit for Osteogenesis (Beyotime, C0148S) following the manufacturer’s protocol. Lastly, the mineralization level of osteoblasts was evaluated based on the intensity of ARS staining.

### *In silico* docking analysis

To elucidate the binding mechanism of KAE to IRE1α, *in silico* docking analysis was performed using AutoDock Suite 4.0 (43). The crystal structure of IRE1α complexed with ADP (PDB ID: 3P23) was chosen as the target protein. Ligands were sourced from PubChem in .sdf format and then converted to .pdb format using Chem 3D 20.0. Polar hydrogen atoms were added to the macromolecule before initiating the docking procedure. Gasteiger charges were assigned to the ligands, and Kollman charges were applied to the receptor molecule using AutoDock Tools software (Version 1.5.7, https://autodock.scripps.edu/).

Throughout the study, the macromolecule was maintained in a rigid state, with rotatable bonds designated for the ligands. The grid was centered on the macromolecule, covering its entire surface area and serving as the search space. The AutoGrid 4.0 software (https://autodock.scripps.edu/) was utilized to generate map files of the flexible atoms, and the docking parameter file was produced using the Lamarckian Genetic algorithm in the AutoDock 4.0 program package. Validation of the docking study was performed by a re-docking experiment using ADP with the solved crystal structure of the kinase domain of IRE1α. Binding energies of the best-docked pose of the docked complexes were calculated considering nonbonded interactions, torsional energy, hydrogen bonding, and desolvation energies, yielding an estimated Free Energy of Binding of −6.00 kcal/mol.

For a comprehensive analysis of the interactions at the 2D level, the LigPlot^+^ software (Version 2.2.8, https://www.ebi.ac.uk/thornton-srv/software/LigPlus/) was employed, while 3D interactions were visualized using the PyMOL molecular graphics system (Version 2.5.4, https://www.pymol.org/).

### qRT-PCR assay

Total RNAs were isolated from cultured cells with TRIzol reagent (Ambion, 15596018). Complementary DNA was synthesized by reverse transcription of 500 ng total RNA using the HiScript Ⅲ qRT SuperMix (Vazyme, R323-01). qRT-PCR was conducted using the ChamQ SYBR qPCR Master Mix (Vazyme, Q341-02/03) on a StepOnePlus Real-Time PCR System (Thermo Fisher Scientific), according to the manufacturer’s instructions. Individual gene expression was normalized to β-actin expression. The primer sequences of the target mRNAs (*Hspa5*, *Ddit3*, *Atf4*, *Bax*, *Bcl2*, *Bloc1s1*, *Scara3*, *Hgsnat*, *Col6α1*, *Pmp22*) were used as indicated in Table S1 (16, 18).

### PCR analysis of XBP1 mRNA cleavage by IRE1α

To detect both spliced and unspliced XBP1 mRNA, previously described mouse XBP1 primers were employed (Forward: 5′-ACACGCTTGGGAATGGACAC-3′, reverse: 5′-CCATGGGAAGATGTTCTGGG-3′) (84). Briefly, complementary DNA was synthesized and then subjected to PCR amplification using the XBP1-specific primers, resulting in the generation of a 171 bp unspliced XBP1 fragment and a 145 bp spliced XBP1 fragment. Subsequently, the PCR products were separated by electrophoresis on a 3% agarose gel stained with ethidium bromide, followed by visualization under UV light using a gel imaging system (Tanon 2500). Uncropped agarose gel images are shown in Figure S8.

### RNA-seq analysis

Total RNA was extracted from primary mouse osteoblasts following treatment with or without TiPs (50 μg/ml) for 24 h. All the RNA-seq and subsequent analyses work were conducted by Huada Gene Company to acquire the Fragments Per Kilobase of transcript per Million mapped reads values for all genes. Analysis of DEGs was carried out using the limma package in the R software environment, employing a significance threshold of *p* < 0.05, and fold changes >1.5 were applied for the analysis. The clean RNA-seq reads have been deposited in the NCBI SRA database (http://www.ncbi.nlm.nih.gov/sra) under the BioProject accession ID: PRJNA1105555.

### Statistical analysis

All data are presented as mean ± SD. Statistical analysis was performed using GraphPad Prism software (Version 8.0.2, https://www.graphpad.com/scientific-software/prism/). Unpaired two-tailed Student’s *t* test was used for comparison between two groups. When comparing more than two groups, the homogeneity of variances was evaluated using the Brown-Forsythe test, and multiple comparisons were conducted using One-way ANOVA followed by Tukey’s *post hoc* tests. A significance level of *p* < 0.05 was considered statistically significant, while *p* < 0.01 was deemed strongly significant.

## Data availability

Data will be made available on request.

## Supporting information

This article contains supporting information.

## Conflicts of interest

The authors declare that they have no conflicts of interest with the contents of this article.
