# Supplementary material for: Kaempferol attenuates particle-induced osteogenic impairment by regulating ER stress via the IRE1α–XBP1s pathway
Source: J Biol Chem. 2024 May 18;300(6):107394. doi: 10.1016/j.jbc.2024.107394 (PMC11223082; doi:10.1016/j.jbc.2024.107394)
Supplement: Supporting Figures S1–S8 and Tables S1 [file mmc1.docx]

**Supplementary Information**

**Kaempferol attenuates particle-induced osteogenic impairment by regulating ER stress *via* the IRE1α-XBP1s pathway**

Xin Yu ^1, ‡^, Zhengrong Ren ^2, ‡^, Yuxiang Wang ^1, ‡^, Guodong Yuan ^3, ‡^, Jianlun Hu ^4^, Lin Song ^2^, Cheng Pan ^5^, Kangkang Feng ^4^, Yuqiao Liu ^6^, Longgang Shao ^7^, Li Zhang ^8^, Jinjuan Wang ^9, *^, Jianning Zhao ^1, *^, Nirong Bao ^1, *^, Zhongyang Sun ^1, 10, *^

**^1^** Department of Orthopedics, Nanjing Jinling Hospital, Affiliated Hospital of Medical School, Nanjing University, Nanjing, China.

**^2^** State Key Laboratory of Pharmaceutical Biotechnology, School of Life Sciences, Nanjing University, Nanjing, China.

**^3^** Department of Orthopedics, The Second Hospital of Nanjing, Nanjing University of Chinese Medicine, Nanjing, China.

**^4^** State Key Laboratory of Pharmaceutical Biotechnology, Jiangsu Key Laboratory of Molecular Medicine, Medical School, Nanjing University, Nanjing, China.

**^5^** Department of Thoracic Surgery, The First Affiliated Hospital of Nanjing Medical University, Nanjing, China.

**^6^** Medical Information Data Bank, Nanjing Jinling Hospital, Affiliated Hospital of Medical School, Nanjing University, Nanjing, China.

**^7^** Department of Emergency Medicine, The Second Affiliated Hospital of Nanjing University of Chinese Medicine, Nanjing, China.

**^8^** Department of Prosthodontics, Nanjing Stomatological Hospital, Affiliated Hospital of Medical School, Nanjing University, Nanjing, China.

**^9^** Department of Pharmacy, Nanjing First Hospital, Nanjing Medical University, Nanjing, China.

**^10^** Department of Orthopedics, Air Force Hospital of Eastern Theater, Anhui Medical University, Nanjing, China.

^‡^ These authors contributed equally to this study.

^*^ For correspondence: Jinjuan Wang; Jianning Zhao; Nirong Bao; Zhongyang Sun

E-mail addresses: 1492636871@qq.com (Jinjuan Wang), zhaojianning.0207@163.com (Jianning Zhao), bnrbnr@sina.com (Nirong Bao), szylpxt@163.com (Zhongyang Sun).

**Fig. S1. Biosafety assessment of TiPs and KAE *in vivo*.** (A) Scanning electron microscopy (SEM) and transmission electron microscopy (TEM) images of TiAl_6_V_4_ particles (TiPs). Scale bar, 500 nm (left), 100 nm (right). (B) Chemical structure, formula, molecular weight, and PubChem CID of KAE. (C) Alternations in mouse body weight change of the mice in each group. *n* = 6. (D) Serum levels of alanine aminotransferase (ALT), aspartate aminotransferase (AST), blood urea nitrogen (BUN), and creatine. *n* = 6. (E) Representative H&E staining images of major organs (heart, liver, spleen, lung, and kidney) harvested from each group. Scale bar, 100 μm. All values are presented as mean ± SD.

**Fig. S2. Effects of TiPs and KAE on osteoblast viability and apoptosis.** (A-B) Flow cytometry analysis of apoptosis levels in osteoblasts exposed to varying concentrations (0, 10, 50, 100, and 200 μg/ml) of TiPs using Annexin V/PI staining. *n* = 3. (C) Cell viability analysis of primary mouse osteoblasts incubated with varying concentrations of TiPs (0, 10, 50, 100, and 200 μg/ml) at different time points (12, 24, and 48 h). (D) Effects of KAE treatment (0, 1, 5, 10, 20, and 40 μM) for 24, 48, and 72 h on cell viability, as assessed by CCK-8 assay. *n* = 3. (E) Osteoblasts treated with TiPs were co-cultured with varying concentrations (0, 1, 5, 10, and 20 μM) of KAE for 12, 24, and 48 h. Cell viability was assessed by CCK-8 assay. *n* = 3. (F-G) The apoptosis rate of osteoblasts was detected by flow cytometry analysis using Annexin V/PI staining in different groups with various concentrations (0, 1, 5, 10, 20 μM) of KAE for 24 h. *n* = 3. All values are presented as mean ± SD. One-way ANOVA. ^*^*P* < 0.05 and ^**^*P* < 0.01 *vs.* the control group. *ns*, not statistically significant *vs.* the control group. ^##^*P* < 0.01 *vs.* the TiPs group.

**Fig. S3.** **KAE alleviated TiPs-induced osteoblast apoptosis by downregulating ER stress-mediated apoptosis signaling *in vitro*.** (A) Western blot analysis of CHOP in osteoblasts following treatment for 24 h with medium, TiPs, or TiPs + KAE. *n* = 3. (B) Western blot analysis of C-CASP3 in osteoblasts following treatment for 24 h with medium, TiPs, or TiPs + KAE. *n* = 3. (C) Western blot analysis of BAX, BCL2, and Cyto C in osteoblasts following treatment for 24 h with medium, TiPs, or TiPs + KAE. *n* = 3. All values are presented as mean ± SD. One-way ANOVA. ^**^*P* < 0.01 *vs.* the control group. ^#^*P* < 0.05 and ^##^*P* < 0.01 *vs.* the TiPs group.

**Fig. S4. KAE** **treatment alone enhanced osteoblast activity *in vitro*.** (A) Primary osteoblasts were treated with varying concentrations (0, 2.5, 5, and 10 μM) of KAE for 24, 48, 72, and 96 h. Cell viability was assessed using the CCK-8 assay. *n* = 3. (B) Representative images of ALP staining and ARS staining for osteoblasts in each group. Scale bar, 5 mm. (C-D) Western blot analysis of osteogenic markers (RUNX2, ALP, and OCN) in osteoblasts treated with varying concentrations (0, 2.5, 5, and 10 μM) of KAE after 3 days of osteogenic induction. *n* = 3. All values are presented as mean ± SD. One-way ANOVA. ^*^*P* < 0.01 and ^**^*P* < 0.05 *vs.* the control group.

**Fig. S5. KAE could enhance IRE1α activation to promote XBP1 mRNA splicing.** (A-D) Molecular docking poses of KAE, ADP, APY29, and IXA4 within the nucleotide-binding site of the IRE1α kinase domain generated using AutoDock Suite 4.0. A superimposed representation was constructed using PyMOL, with dashed yellow lines representing hydrogen bonding interactions. (E) Western blot analysis of IRE1α, p- IRE1α, and XBP1s in osteoblast following treatment with KAE alone for 0, 4, 8, 12, and 24h, respectively. *n* = 3. (F) qRT-PCR analysis of RIDD substrate (*Bloc1s1*, *Scara3*, *Hgsnat*, *Col6α1*, *Pmp22*) mRNA levels in osteoblasts following treatment with KAE alone for 24 h. *n* = 3. (G) Western blots of JNK and p-JNK in osteoblast following treatment with KAE alone for 0, 4, 8, 12, and 24h, respectively. All values are presented as mean ± SD. One-way ANOVA. ^*^*P* < 0.05 and ^**^*P* < 0.01 *vs.* the control group.

**Fig. S6.** **IRE1α/XBP1s inhibition with STF-083010 abrogated the protective effects of KAE on osteoblasts exposed to TiPs *in vitro*.** (A) Western blot analysis of XBP1s in osteoblasts following treatment for 24 h with or without TiPs, KAE, and STF-083010. *n* = 3. (B) Western blot analysis of CHOP in osteoblasts following treatment for 24 h with or without TiPs, KAE, and STF-083010. *n* = 3. (C) Western blot analysis of C-CASP3 in osteoblasts following treatment for 24 h with or without TiPs, KAE, and STF-083010. *n* = 3. All values are presented as mean ± SD. One-way ANOVA. ^**^*P* < 0.01 *vs.* the control group. ^##^*P* < 0.01 *vs.* the TiPs group. ^&&^*P* < 0.01 *vs.* the TiPs + KAE group.

**Fig. S7. IRE1α/XBP1s inhibition with STF-083010 abrogated the protective effects of KAE on stressed osteoblasts in the mouse model of TiPs-induced osteolysis.** (A-B) Quantitative measurement of EBS/BS (%) and BT (μm) in each group. *n* = 6. (C-D) Quantitative immunohistochemical analysis for OCN and COL1α1 in each group. *n* = 6. (E) Quantitative measurement of the average periosteum mineral apposition rates (MAR, μm/d) in each group. *n* = 6. All values are presented as mean ± SD. One-way ANOVA. ^**^*P* < 0.01 *vs.* the control group. ^#^*P* < 0.05 and ^##^*P* < 0.01 *vs.* the TiPs group. ^&^*P* < 0.05 and ^&&^*P* < 0.01 *vs.* the TiPs + KAE group.

**Fig. S8. Uncropped Western blots and Agarose gels.**

| Mouse gene | Forward primers (5’-3’) | Reverse primers (5’-3’) |
| --- | --- | --- |
| *Hspa5* | GCATCACGCCGTCGTATGT | ATTCCAAGTGCGTCCGATGAG |
| *Ddit3* | CTGGAAGCCTGGTATGAGGAT | CAGGGTCAAGAGTAGTGAAGGT |
| *Atf4* | CTCTTGACCACGTTGGATGAC | CAACTTCACTGCCTAGCTCTAAA |
| *Bax* | AGACAGGGGCCTTTTTGCTAC | AATTCGCCGGAGACACTCG |
| *Bcl2* | GCTACCGTCGTGACTTCGC | CCCCACCGAACTCAAAGAAGG |
| *Bloc1s1* | TCCCGCCTGCTCAAAGAAC | GAGGTGATCCACCAACGCTT |
| *Scara3* | TGACAGGGATGTACTGTGTGT | TGCAAAGATAGGTTCTTCTGGC |
| *Hgsnat* | CCAGTGGACAGTAACCTTCCT | CAGAATTGATGAGACGGTCAGTT |
| *Col6α1* | GGATCTATTCTTCGTGCTCGAC | TCTCAGGTTGTCAATGAAGCG |
| *Pmp22* | CATCGCGGTGCTAGTGTTG | GATCAGTCGTGTGTCCATTGC |
| *β-actin* | GTGACGTTGACATCCGTAAAGA | GCCGGACTCATCGTACTCC |

**Table S1. The primer sequences used for qRT-PCR.**
